# Discovery of Active Ingredients Targeted TREM2 by SPR Biosensor-UPLC/MS Recognition System, and Investigating the Mechanism of Anti-Neuroinflammatory Activity on the Lignin-Amides from *Datura metel* Seeds

**DOI:** 10.3390/molecules26195946

**Published:** 2021-09-30

**Authors:** Si-Yi Wang, Yan Liu, Xiao-Mao Li, Adnan Mohammed Algradi, Hai Jiang, Yan-Ping Sun, Wei Guan, Juan Pan, Hai-Xue Kuang, Bing-You Yang

**Affiliations:** Key Laboratory of Chinese Materia, Heilongjiang University of Chinese Medicine, Ministry of Education, Harbin 150040, China; WSY06182021@163.com (S.-Y.W.); lifeliuyan@163.com (Y.L.); 18216074841@163.com (X.-M.L.); dradnan86@hotmail.com (A.M.A.); JiangHai_777@126.com (H.J.); 18704608056@163.com (Y.-P.S.); myguanwei1234@yeah.net (W.G.); panjuan1002@hotmail.com (J.P.)

**Keywords:** TREM2, SPR, seeds of *Datura metel*, lignin-amides, neuroinflammation

## Abstract

As a new target protein for Alzheimer’s disease (AD), the triggering receptor expressed on myeloid Cells 2 (TREM2) was expressed on the surface of microglia, which was shown to regulate neuroinflammation, be associated with a variety of neuropathologic, and regarded as a potential indicator for monitoring AD. In this study, a novel recognition system based on surface plasmon resonance (SPR) for the TREM2 target spot was established coupled with quadrupole time-of-flight tandem mass spectrometry (UPLC-MS), in order to screen the active ingredients targeting TREM2 from *Datura metel* seeds. The results showed that four lignan-amides were discovered as candidate compounds by SPR biosensor-UPLC/MS recognition analysis. According to the guidance of the active ingredients discovered by the system, the lignin-amides from *Datura metel* seeds (LDS) were preliminarily identified as containing 27 lignan-amides, which were enriched compositions by the HP-20 of *Datura metel* seeds. Meanwhile, the anti-inflammatory activity of LDS was evaluated in BV2 microglia induced by LPS. Our experimental results demonstrated that LDS could reduce NO release in LPS-treated BV2 microglia cells and significantly reduce the expression of the proteins of inducible Nitric Oxide Synthase (iNOS), cyclooxygenase 2 (COX-2), microtubule-associated protein tau (Tau), and ionized calcium-binding adapter molecule 1 (IBA-1). Accordingly, LDS might increase the expression of TREM2/DNAX-activating protein of 12 kDa (DAP12) and suppress the Toll-like receptor SX4 (TLR4) pathway and Recombinant NLR Family, Pyrin Domain Containing Protein 3 (NLRP3)/cysteinyl aspartate specific proteinase-1 (Caspase-1) inflammasome expression by LDS in LPS-induced BV2 microglial cells. Then, the inhibitory release of inflammatory factors Interleukin 1 beta (IL-1β), Interleukin 6 (IL-6), and Tumor necrosis factor-alpha (TNFα) inflammatory cytokines were detected to inhibit neuroinflammatory responses. The present results propose that LDS has potential as an anti-neuroinflammatory agent against microglia-mediated neuroinflammatory disorders.

## 1. Introduction

AD is a progressive neurodegenerative disease with high incidence and severe economic burden. Microglial activation is one of the major pathological signs [1,2]. At the same time, neuroinflammation is closely related to AD, which is mediated by the activation of microglia [3]. One of the key molecules involved in the activation of small cells is TREM2, a cell surface receiver of the Ig superfamily. TREM2 and DAP12 are bound to produce complexes, which can regulate the phagocytosis of apoptotic neurons and inhibit the neuroinflammatory effect by regulating the inflammatory pathway [4,5,6,7]. In addition, it has been shown that a lack of TREM2 will lead to an Aβ prominent phenomenon [8,9]. In recent studies, the level of TREM2 as a new predictive biomarker for the occurrence of AD may have clinical significance [10,11]. Therefore, TREM2 has attracted lots of attention as a new target. Hence, we first established the SPR-UPLC-MS/MS system that targeted TREM2 protein for sifting the active ingredients from herbal medicine. The seeds of *Datura metel* had an intensive response signal in the SPR system targeted at TREM2, and four lignin-amides were identified that were regarded as TREM2 targeted active candidates by UPLC-MS analysis. Coincidentally, the lignan-amides demonstrated outstanding effects for AD treatment involving acetylcholinesterase inhibitory activity, anti-oxidation, and anti-inflammatory in previous modern pharmacological studies [12,13,14,15,16]. Simultaneously, the rich lignin-amides existed in the seeds of *Datura metel* including cannabisin D-H [17,18,19,20], and it possesses the traditional effects of the treatments of convulsion, epilepsy, and rheumatic arthralgia according to the Compendium of Materia Medica [3], together with analgesic, hypoglycemic, and anti-inflammation biological activities [21,22,23]. However, it should be noted that the underlying molecular mechanism remains unclear and needs further exploration, although some studies have indicated the anti-neuroinflammatory potential of LDS. 

In this study, the TREM2 targeted SPR-UPLC-MS active ingredients recognition system was firstly established by determining optimal pH and the amount of protein immobilization for TREM2 coupling. Simultaneously, four lignan-amides were discovered and characterized from *Datura metel* seeds. Being limited to the components obtained by the SPR system, LDS were enriched by HP-20 macroporous resin and identified as covering 27 compounds, so as to perform more in-depth research and development. In this context, this was the first work to evaluate the capacity of LDS to inhibit neuroinflammation induced by LPS in BV2 microglial cells, which was regulated by the TREM2/DAP12, TLR4 pathway and NLRP3/Caspase-1 inflammasome expression. Thus, the possible mechanism of LDS in the treatment of anti-neuroinflammatory was discussed, and our research elucidated the diversity of anti-neuroinflammatory mechanisms exerted by a natural product.

## 2. Results and Discussion

### 2.1. Construction of SPR Biosensor-UPLC/MS Active Ingredient Recognition System for TREM2 Target

#### Immobilization of TREM2 on SPR Active Ingredients Recognition System

**Figure 1:**
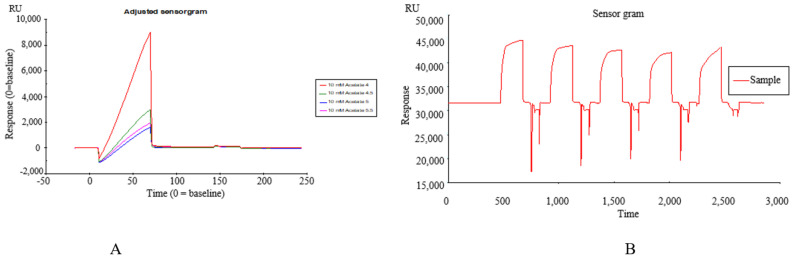
(**A**) The pre-coupling of TREM2 protein at different pH values of 4.0, 4.5, 5.0, 5.5; (**B**) The binding affinity of *Datura metel* seeds to TREM2 by SPR assay.

TREM2 was diluted in 10 mM sodium acetate (pH 4.0) that was determined by a pre-coupling experiment and immobilized by the amine coupling method on a CM5 sensor chip according to the manual-injections protocol. At the same time, 4 flow cells of the chip were selected for sample loading. Definitively, the target immobilization level of the TREM2 was 5000 RU (Figure 1A,B).

### 2.2. Identification of TREM2 Target Direct Binding Active Ingredients

**Figure 2:**
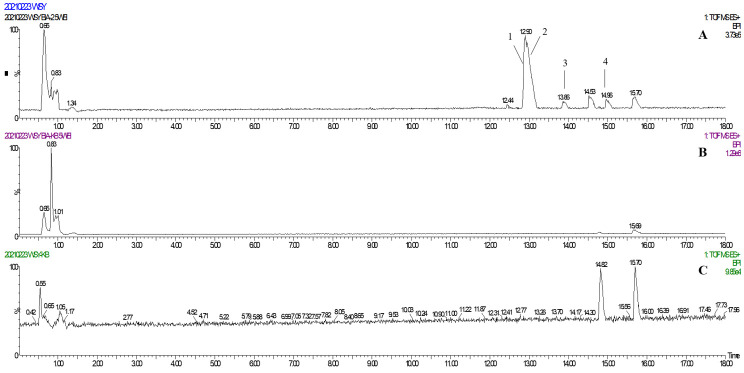
(**A**) The base peak chromatogram of the TREM2 bound ingredients from *Datura metel* seeds; (**B**) The base peak chromatogram of the negative control experiment for SPR assay; (**C**) The base peak chromatogram of the solvent blank.

**Figure 3:**
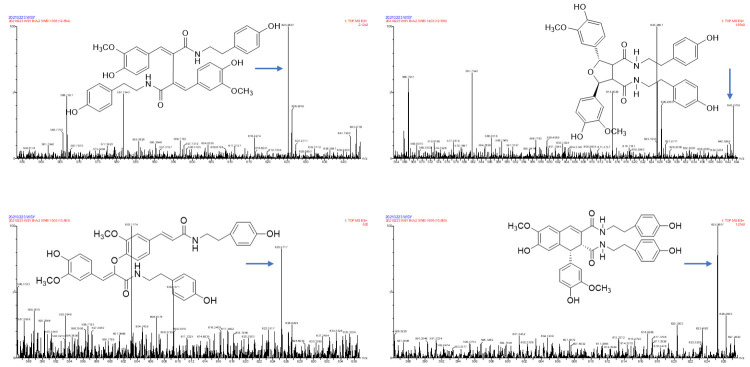
Identification of the TREM2 bound ingredients from *Datura metel* seeds.

**Figure 4:**
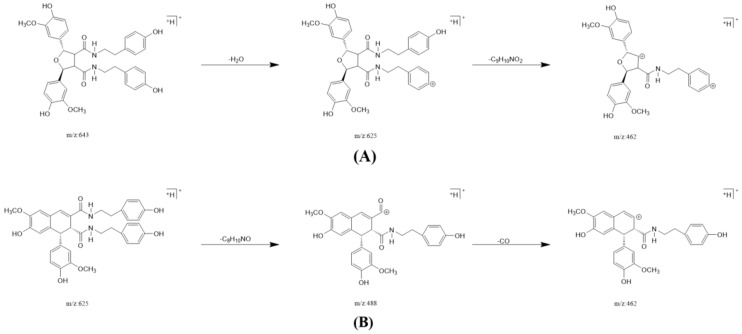
The possible fragment pathways of the TREM2 bound ingredients from *Datura metel* by using UPLC-QTOF-MS/MS. (**A**) lyciumamide K; (**B**) cannabisin D.

In one single injection and recovery procedure, the amount of chip-bound ingredients was quite low and insufficient to meet the detection limit of QTOF/MS. A series of sample vials were obtained by repeated injection and recovery operations. Then, the different numbers of the vials were merged, dried under nitrogen, redissolved by mobile phase, and analyzed by UPLC-QTOF/MS system. According to the accurate mass data obtained by QTOF/MS and searching the chemical compound database of the components containing compounds built by our group, only cannabisin G (peak 1), lyciumamide K (peak 2), cannabisin F (peak 3), and cannabisin D (peak 4) were identified as the recovered ingredients (Figure 2 and Figure 3 and Table 1). Lyciumamide K was formed by the connection of hydroxyl cinnamic acid and purecine, the mass spectroscopic cracking pattern of which was a C-N bond fracture and α-elimination [24]. Its excimer ion peak was 643 [M+H]^+^ and its fragment ion were 625 and 462, which may be related to the loss of one fragment of H_2_O and C_10_H_11_NO_2_, respectively (Figure 4A). Among them, cannabinoid G, cannabinoid F, and cannabinoid D are all formed by the connection of lignans and two aromatic amines, which were confirmed to pass the comparison of the standard product (Figures S1–S3). The cannabinoid D was chosen as an example, and its excimer ion peak was 625 [M+H]^+^ while its fragment ion were 488, 462, which may be related to the neutral loss of C_8_H_10_NO and C_9_H_10_NO_2_, respectively (Figure 4B). 

### 2.3. Annotation of the Enriched Ingredients LDS by UPLC-QTOF/MS Technique

**Figure 5:**
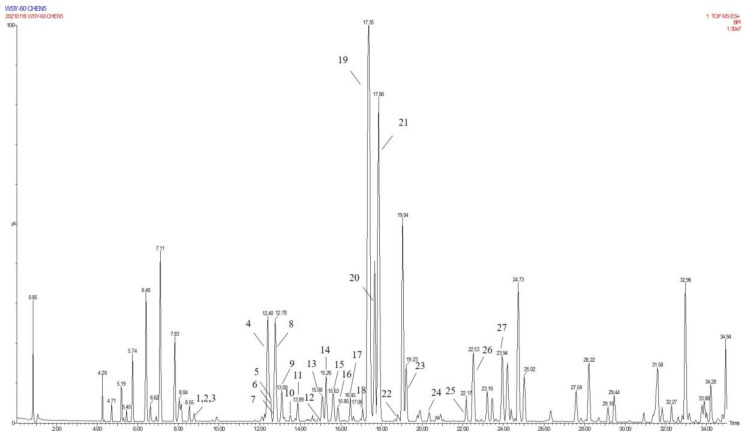
The base peak of LDS in UPLC-QTOF mass spectrometry.

**Figure 6:**
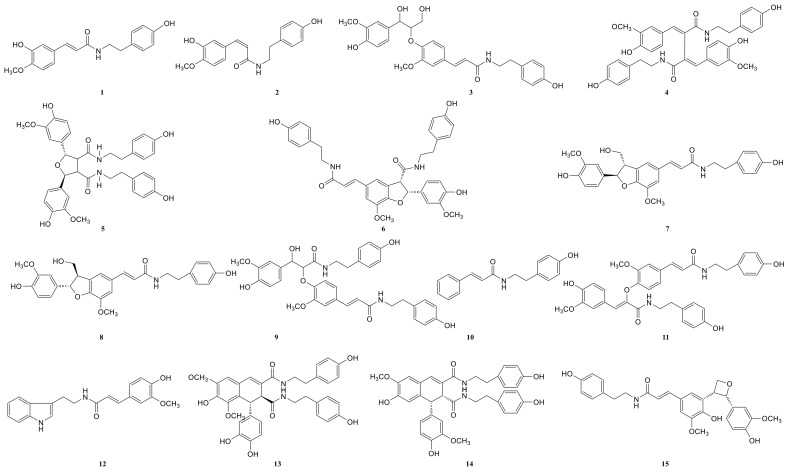

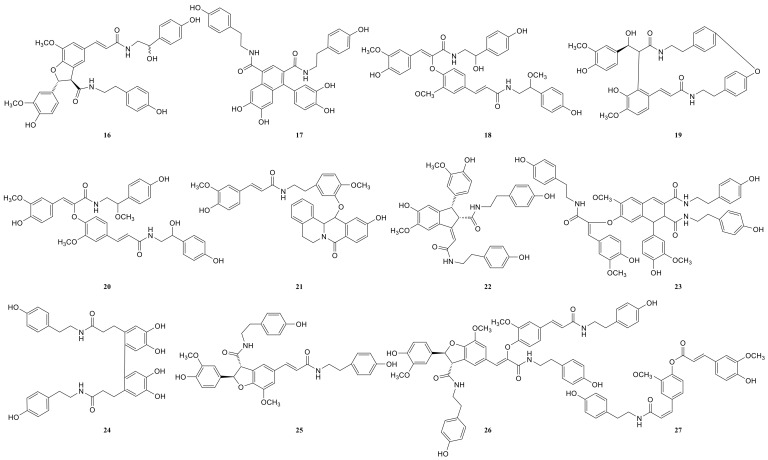
The structures of lignan-amides identified from LDS.

Due to the small sample size of TREM2-bounding active ingredients, we could not meet the needs of a further experimental study of its mechanism. Therefore, the enrichment of lignan-amides in the seeds of *Datura metel* were conducted in this study. The results were as follows. The data obtained from the UPLC-QTOF/MS analytical technique annotated 27 lignan-amides (Table 2, Figure 5 and Figure 6) experiments (MS/MS) support the formation of fragment ions which were crucial to ensure greater reliability to authenticate the metabolites by comparison with the literature. The structures which could considered were formed by connecting phenylpropane and amine fragments through amide bonds [12,17,20,25,26,27,28,29,30,31,32]. 

Peaks 1 and 2 gave [M+H]^+^ ion at *m*/*z* 314.1388 with a molecular formula C_18_H_19_NO_4_, and produced fragment ions at *m*/*z* 177 [M+H-C_8_H_10_NO]^+^, which were presumed as N-trans-feruloyl tyramine, N-cis-feruloyl tyramine. Peak 3 gave [M+H]^+^ ion at *m*/*z* 510.2124 and fragment ions at *m*/*z* 312 [M+H-H_2_O-3CH_3_COOH]^+^, which indicated that it could be presumed as cannabisin H. It was identified by comparison to a standard product (Figure S1). The peaks 4, 6, 11, 14, 19, 22, and 25 were deemed as cannabisin G, Tribulusamide A, cannabisin F, cannabisin D, (2aS,3aS) lyciumamide D, and grossamide, which displayed [M+H]^+^ ion at *m*/*z* 625 and produced predominant fragment ions at 462 due to losses of [C_9_H_10_NO_2_] fragments. 

For peak 5, except for the fragment ions 643 [M+H]^+^, the characteristic intermediate ions [M+H-H_2_O]^+^ at *m*/*z* 625 and [M+H-H_2_O-C_10_H_11_NO_2_]^+^ at *m*/*z* 462 from peak 5, which suggested the compound were lyciumamide K. The fragment ions at *m*/*z* 492 [M+H]^+^ and 462 [M+H-OCH_3_]^+^ corresponded with successive neutral losses of methoxy group residue, indicating that peaks 7, 8, and 15 were grossamide K, cis-grossamide K, and lyciumamide C, respectively. Peak 9 gave [M+H]^+^ at *m*/*z* 643.2657, which further fragmented into *m*/*z* 462 [M+H-H_2_O-C_9_H_11_NO]^+^, so their fragment ions suggested that it was cannabisin E. Peak 10, determined as N-trans-cinnamoyl tyramine, showed [M+H]^+^ at *m*/*z* 268.1342, and fragment ion at *m*/*z* 177, corresponding to missing of 103 Da. Peak 12, determined as N-trans-ferulyl tryptamine, showed [M+H]^+^ at *m*/*z* 337.1552, and fragment ion at *m*/*z* 177 [M+H-C_10_H_11_N_2_]^+^. The characteristic intermediate ions [M+H]^+^ at *m*/*z* 641 and [M+H-5H_2_O-2HOCH_3_-C_8_H_9_]^+^ at *m*/*z* 323 from peak 13 suggested it was (1R)-N, N'-Bis(4-hydroxyphenethyl)-1*β*-(3,4-dihydroxyphenyl)-6,8-dimethoxy-7-hydroxy-1,2-dihydronaphthalene-2*α*,3-dicarboxamide. Peak 16 was determined as Melongenamide B, which had fragment ion at *m*/*z* 625 [M+H-H_2_O]^+^ and 537 [M+H-3H_2_O-CH_3_COOH]^+^. Peak 17, determined as cannabisin A, showed [M+H+CH_3_COOH]^+^ at *m*/*z* 656 and MS/MS fragment ions at *m*/*z* 494 correspondings to the missing of the fragment [C_9_H_10_NO_2_]. Peaks 18 and 20 were determined as Melongenamide F ([M+H]^+^ at *m*/*z* 671) and Melongenamide G ([M+H]^+^ at *m*/*z* 671), respectively. Both constituents had fragment ions at *m*/*z* 437 (loss of 3H_2_O, HOOCH_3_, and C_10_H_4_). Chenoalbicin gave [M+H+H_2_O]^+^ ion at *m*/*z* 625.2550, which was observed in peak 21. Peak 23 displayed [M+H]^+^ ion at *m*/*z* 936.3699 ([M+H]^+^) and produced predominant fragment ions at 625 [M+H-C_18_H_17_NO_4_]^+^. The compound was referred to as Thoreliamide C. Peak 24 was assigned as lyciumamide F derivative based on the fragment ions at *m*/*z* 623.2397 ([M+Na]^+^). Peak 26 gave [M+H]^+^ ion at *m*/*z* 936.3713 and fragment ions at *m*/*z* 599 [M+H-H_2_O-CH_3_OH-CO-C_17_H_17_NO_2_]^+^, which indicated that it was presumed as cannabisin O. Peak 27, with deprotonated molecule ion [M+H+HCOOH]^+^ at *m*/*z* 538, was characterized as hibiscuwanin B by the existence of the fragment ions at *m*/*z* 439 (missing of two CH_3_OH groups and two water molecule). 

Presently, only four lignan amides have been identified from the extract of *Datura metel* seeds, which were based on the SPR biosensor-UPLC/MS active ingredients recognition system for the TREM2 target spot. Some of the other lignan amides also showed good anti-neuroinflammatory activity. Most of them showed effects on memory dysfunction and biomarkers of neuroinflammation in the literature, such as cannabisin E (peak 9), N-trans-ferulyl tryptamine (peak 12), cannabisin A (peak 17), cannabisin O (peak 26), and grossamide (peak 25) [29,30,33]. For instance, studies have shown that N-trans-ferulyl tryptamine, cannabisin A, cannabisin H, grossamide K, cannabisin E, and other compounds which can induce apoptosis, inhibit autophagy, and significantly inhibit the proliferation of U-87 cancer cells. Meanwhile, the complex of cannabisin E (peak 9), cannabisin D (peak 14), cannabisin A (peak 17), and grossamide (peak 25) could significantly improve the learning and memory of LPS-induced neuro-inflammatory mice and it significantly improved their cognitive function. Moreover, the low-dose significantly reduced the expression of IL-1β, IL-6, and TNF-α in the brains of LPS-induced mice. According to the literature, cannabisin D (peak 9) and N-trans-ferulyl tryptamine (peak 12) exhibited powerful DPPH• radical-scavenging activity ranging from 69.1% to 86.9% at the concentration of 100 μg/mL. Cannabisin G (peak 4) exhibited significant inhibitory effects on TNF-α release from LPS-induced BV2 microglia cells. Moreover, anti-neuroinflammatory effects of grossamide (peak 25) suppression of TLR-4/MyD88-mediated NF-κB signaling pathways in LPS stimulated BV2 microglia cells. In total, further study still needs to be performed to elucidate the anti-neuroinflammatory contribution of *Datura metel* seeds.

### 2.4. The LDS Inhibited LPS-Induced NO Release in BV2 Cells

**Figure 7:**
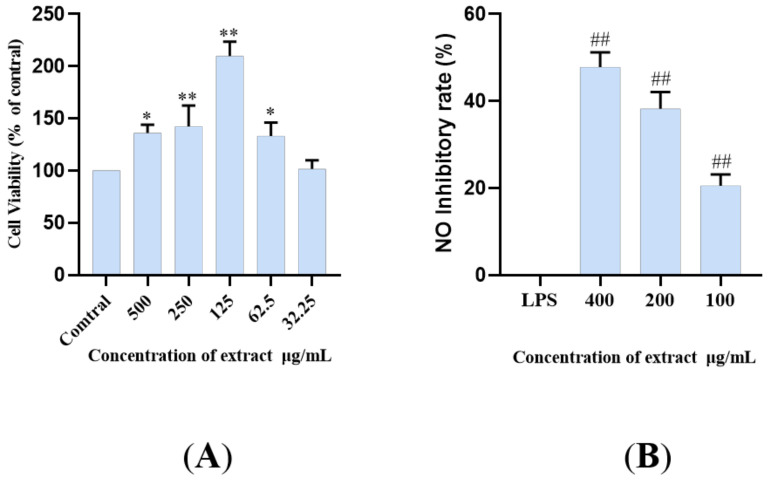
Cell viability (% of cell control) of BV2 cell line treated with LDS according to CCK-8 assay (**A**). Inhibition of NO release in BV2 cells by the LDS (**B**). Values were expressed as mean ± SEM with at least three independent experiments. * *p* < 0.05, ** *p* < 0.01 vs. Control group; ^##^ *p* < 0.01 vs. Model group.

LPS, as an endotoxin, can induce inflammation by increasing the sensitive inflammatory pathway, activating and accumulating inflammatory molecules, thus promoting the production and release of NO (LPS+BV2). The BV2 microglia model induced by LPS is used as one of the commonly used cell models. The principal pathways of inflammation include TLR4/MyD88, NLRP3/Caspase1, and TREM2/DAP12 pathways, and so on [7,9,12,34,35,36]. In this study, an LPS induced microglia inflammatory cell model was used to study the inhibitory effect of lignan-amides in *Datura metel* seeds on the release of inflammatory factor NO. In order to avoid possible toxicity to biological systems caused by the use of natural bioactive compounds, the CCK-8 assay was used to test the cell viability, and the results showed that LDS (50–500 μg/mL) did not significantly reduce the viability of BV2 cells (Figure 7A). Therefore, 400, 200, and 100 μg/mL were selected for subsequent studies. The results showed that the LPS treatment model group induced an increase in NO concentration, while all the treatment groups significantly inhibited the release of inflammatory cytokine NO in BV2 cells (Figure 7B). The LDS may inhibit LPS excessive production of NO by down-regulating iNOS and COX-2 expression, as well as the inhibition of IL-1β, TNFα, and IL-6.

### 2.5. Protein Expression in BV2 Microglia Cells

**Figure 8:**
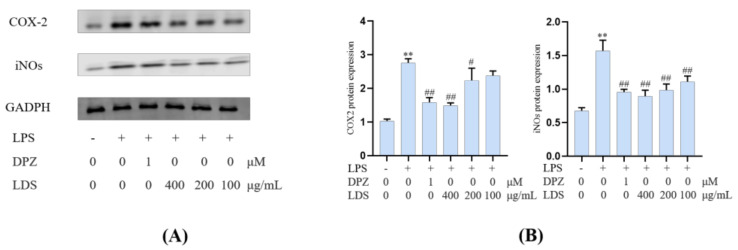
The regulations of microglial cell inflammation of COX—2 and iNOS by LDS. (**A**) Western blotting bands. (**B**) Quantitative data on western blotting bands. Values were expressed as mean ± SEM with at least three independent experiments. ** *p* < 0.01 vs. Control group; ^#^ *p* < 0.05, ^##^ *p* < 0.01 vs. Model group.

Both iNOS and COX-2 were major inflammation-related enzymes involved in the production of NO and prostaglandins. The results showed that the LDS inhibited LPS excessive production of NO by downregulating of iNOS and COX-2 (Figure 8). The iNOS expressed in activated microglia cells played primary roles in NO production of CNS response to various stimuli, including LPS. iNOS was overexpressed in microglia and was likely to mediate programmed cell death in brain injury. The COX-2 protein was an inducible pro-inflammatory molecule. The results indicated that the DPZ and LDS could significantly inhibit NO production and down-regulate protein expression levels of iNOS and COX-2, which suggested that LDS acted to reduce NO production by interfering with TREM2/Dap12 and TLR4 pathways in LPS-stimulated BV2 microglia.

**Figure 9:**
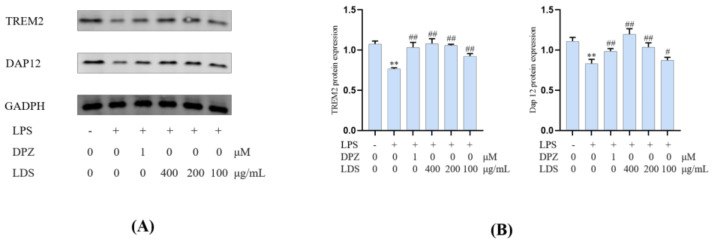
The regulations of microglial cell inflammation of TREM2 and DAP12 by LDS. (**A**) Western blotting bands. (**B**) Quantitative data on western blotting bands. Values were expressed as mean ± SEM with at least three independent experiments. ** *p* < 0.01 vs. Control group; ^#^ *p* < 0.05, ^##^ *p* < 0.01 vs. Model group.

**Figure 10:**
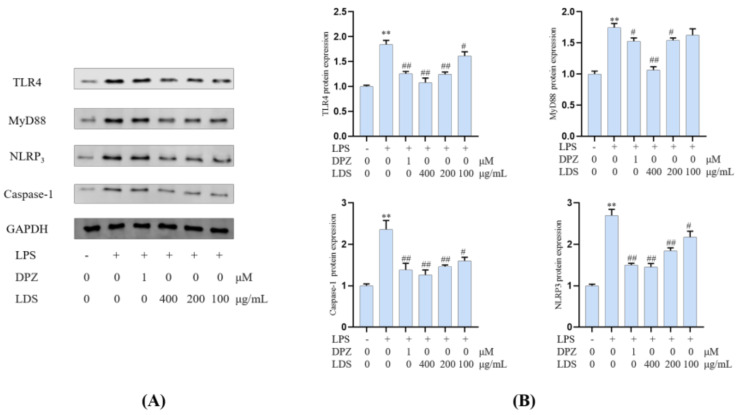
Effects of the LDS on protein expression, including TLR4, MyD88, NLRP3, and Caspase—1 in the BV2 cells of LPS—induced. (**A**) Western blotting bands. (**B**) Quantitative data on western blotting bands. Values were expressed as mean ± SEM with at least three independent experiments. ** *p* < 0.01 vs. Control group; ^#^ *p* < 0.05, ^##^ *p* < 0.01 vs. Model group.

Essential gene expression for two inflammation-related pathways and two proteins, including the TREM2/DAP12 pathway, TLR4 pathway, Tau, and IBA-1, were investigated using Western blotting expression. TREM2 is an avital receptor of microglia, and DAP12 forms a complex with TREM2 through the electrostatic potential across the membrane. Interestingly, we found that LDS treatment dose-dependently increased the expression of TREM2/DAP12 at the protein levels, as we have shown in Figure 8 and Figure 9, suggesting the possible involvement of TREM2/DAP12 in the effects of LDS. As shown in Figure 5, we further examined the expression of main proteins, components in the TLR4 pathway, and NLRP3/Caspase-1 inflammasome expression involved in the production of inflammation-related in BV2 cells. The cells after LPS challenging showed higher expression of TLR4 (Figure 10), MyD88, NLRP3, Caspase-1, than those of the control group. Of importance, treatment with LDS obviously reduced the protein expression of Caspase-1, NLRP3, TLR4, and MyD88, consequently regulating the expression of arthritis factors. Moreover, the bioassay results displayed that the neuroinflammation of LPS stimulated mouse microglia BV2 cells was inhibited, which may be related to the upregulation of protein TREM2/DAP12 leading to the downward expression of TLR4/MyD88, NLRP3/Caspase1 pathway protein. We found that DPZ treatment increased the expression of TREM2/DAP12 and declined the expression in the TLR4 pathway and NLRP3/Caspase-1 inflammasome expression at the protein level. It can be speculated that the anti-inflammatory effect of TREM2 was correlated with the existence of TLR4-associated inflammatory.

**Figure 11:**
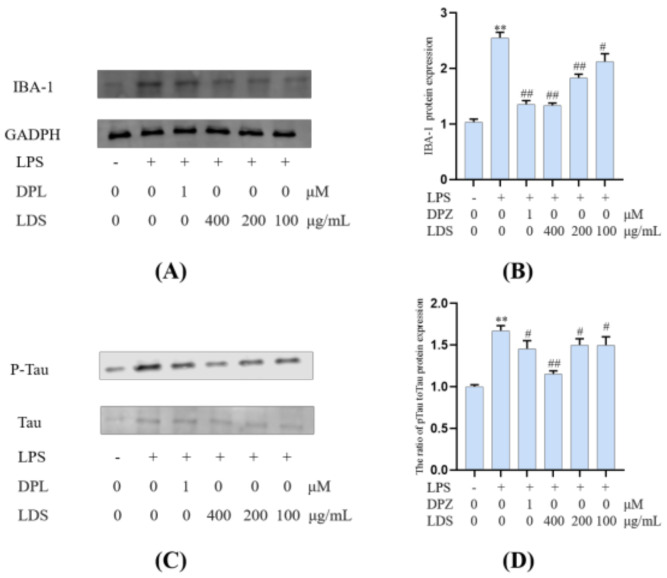
Inhibitory effects on the levels of IBA-1 and the ratio of p—Tau to Tau of LDS. (**A**,**C**) Western blotting bands. (**B**,**D**) Quantitative data on western blotting bands. Values were expressed as mean ± SEM with at least three independent experiments. ** *p* < 0.01 vs. Control group; ^#^ *p* < 0.05, ^##^ *p* < 0.01 vs. Model group.

Microglia constitute the main macrophage in the CNS, and their number and activation degree increase during aging [37,38]. Meanwhile, the activation of microglia was observed in the brain of AD patients, and the presence of Tau protein (Tau-2, Tau-66) in the microglia was also observed in the brain of AD patients [39]. LPS-induced activation of microglia could exacerbate major neuropathological changes in AD, such as the formation of neurofibrillary tangles [40,41]. In addition to neurons, tau protein was also observed in microglia [42,43,44,45,46]. Tau protein was a kind of microtubule-related proteins. When AD occurs, Tau protein was abnormally phosphorylated, and the over-phosphorylated Tau protein loses the microtubule-binding ability and tends to aggregate, forming insoluble helical filaments, leading to neuronal fiber tangles, cytoskeletal abnormalities, and cell death [6]. Our results suggested that the LDS reduced the expression of phosphorylated Tau by promoting the activation of TREM2/DAP12 [47]. This may be related to weakening the expression of NLRP3 and slowing down the inflammation induced by LPS. As a marker of microglia, the protein expression of IBA-1 (Figure 11) was increased in the LPS-induced microglia inflammatory cell model, whereas the expression of IBA-1 was significantly decreased in the LDS group, which may be related to the ascending expression of TREM2/DAP12 and anti-inflammatory activity.

### 2.6. Modulation of Cellular Production of IL-6, IL-1β and TNF-α by the LDS Extract

**Figure 12:**
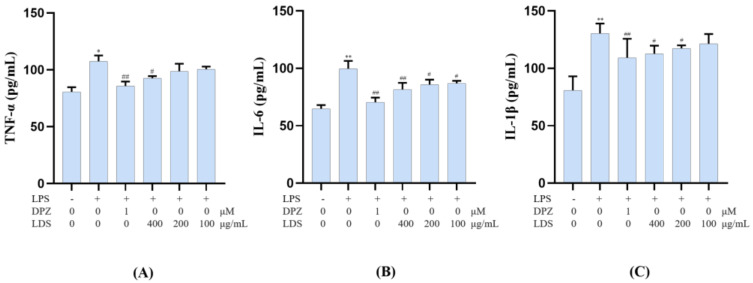
Effects on inflammatory factor TNF-α (**A**), IL-6 (**B**), and IL-1β (**C**) of LDS in the BV2 cells of LPS—induced. Values were expressed as mean ± SEM with at least three independent experiments. * *p* < 0.05, ** *p* < 0.01 vs. Control group; ^#^ *p* < 0.05, ^##^ *p* < 0.01 vs. Model group.

To confirm the anti-neuroinflammation potential of the LDS extract, the release of the pro-inflammatory cytokines IL-6, IL-1β, and TNF-α in the cultured medium was investigated by ELISA kits. However, this LPS-stimulated production of TNF-α, IL-6 and IL-1β were significantly down-regulated by the treatment of the LDS extract (Figure 12A–C), which meant the neuroinflammation of BV2 microglial cells could be suppressed by the effective concentration of the LDS extract and the DPZ. TNF-α was a tumor necrosis factor, which could promote T cells to produce a variety of inflammatory factors and promote the occurrence of inflammatory response. IL-6 was a pleiotropic cytokine playing a central role in inflammation, which was a signal transduction activator and transcriptional activator. IL-1β, also known as a leukocyte heat source, was an important part of inflammatory and closely related to Alzheimer’s disease. These expressions and releases were closely related to the expression of TLRs, NLRP3/Caspase1 influence. We speculated that the down-regulation of the above factors might be related to the activated expression of TREM2/DAP12 and the reduced expression of the TLR4 pathway by the LDS extract.

## 3. Materials and Methods

### 3.1. Materials

The seeds of *Datura metel* were collected in Lingao county, Hainan province. The plant was identified by Professor Rui-Feng Fan of the Heilongjiang University of Chinese Medicine, and its voucher specimen (NO. 20180827) had been deposited at Heilongjiang University of Chinese Medicine. 

Mouse microglial BV2 cell was purchased from the Institute of Cells, Chinese Academy of Science (Shanghai, China). The recombinant protein TREM2 was purchased from Acro (Beijing, China).

### 3.2. Chemical, Reagents, Equipment

DMEM was purchased from Hyclone (Beijing, China). Fetal bovine serum (FBS) was purchased from BI (Israel). Griess reagent was purchased from Beyotime (Shanghai, China). LPS, RIPA buffer, and CF3COOH (TFA) were purchased from Sigma-Aldrich (St. Louis, MO, USA). Rabbit antibodies against mouse iNOS, COX-2, Tau, p-Tau, IBA-1 TREM2, DAP12, TLR4, MyD88, Caspase-1, and NLRP3 were obtained from Abclonal (Wuhan, China). HPLC-grade methanol and acetonitrile were supplied from Merck KGaA (Darmstadt, Germany). PBS, CM5 sensing chips were obtained from from GE Healthcare (Sweden). HP-20 macroporous resin was purchased from Mitsubishi Chemical Company (Japan).

An ultrasonic cleaner, high-speed freezing centrifuge, and electric heater were obtained from Branson (EYELA, Tokyo, Japan), and Biacore T200 (GE Healthcare, Sweden). The Xevo G2-XS Qtof was acquired from Waters Inc (Milford, USA.). Deionized water was collected from a Milli-Qwater purification system (Merck KGaA, Darmstadt, Germany). The rotavapor tandem cold trap was from EYELA (EYELA, Tokyo, Japan). The Epoch 2 microplate reader was got by BioTek (USA). The Electrophoresis Device and scanning imager were acquired from BIO-RAD.

### 3.3. Sample Preparation

In our previous study, we found that the optimized extraction procedure of the *Datura metel* seeds was 95% ethanol (ethanol: water at 95:5) for 90 min with heat-reflux, three times. The seeds of *Datura metel* (12 kg) were extracted according to the above method. The combined EtOH extract was concentrated to yield a residue (2.1 kg) under reduced pressure. 

The extract of the *Datura metel* seeds was dissolved in 5 μL of DMSO to make a basic solution, and then Phosphate-buffered saline (PBS) was used to prepare the sample to be tested in the SPR experiment containing 5% DMSO. Then, the extract was centrifuged, and the supernatant was filtered through a 0.22-μm nylon filter.

### 3.4. Construction of SPR Biosensor-UPLC/MS Active Ingredient Recognition System for TREM2 Target

#### 3.4.1. TREM2 Protein Pre-Coupling

In order to find the optimal equivalent point of the protein, more proteins could be fixed on the chip surface, and different pH values of sodium acetate were optimized. Hence, 100 mg/mL TREM2 was diluted to 20 mg/mL in sodium acetate of different pH (4.0, 4.5, 5.0, 5.5). The flow cell 2 was the detection cell. Then, samples were injected into the CM5 sensor surface for 120 s at a flow rate of 20 μL/min. The sensor-grams were recorded and analyzed by the BiacoreT200 system.

#### 3.4.2. Recovery of TREM2-Bound Ingredients

The ingredients bound to TREM2 on the sensor surface were recovered by an injection and recovery program using the BiacoreT200 system. At the same time, a negative control experiment was established, in which the sample flows through 4 channels of a blank CM5 chip without binding TREM2 protein. Subsequently, the sensor-grams of *Datura metel* seeds were recorded and analyzed. Briefly, the extract was injected over the sensor surface for 180s at 5 μL/min and washed with distilled water. The TFA (2 μL) was injected into the flow cells and incubated for 20 s to allow the bound ingredients to dissociate into the recovery solution. Then, the flow direction over the sensor surface was reversed, and the recovered solution containing TREM2 bound ingredients was deposited in 10 μL ammonium bicarbonate (NH_4_HCO_3_, 50 mM). Eventually, the obtained TREM2 bound ingredients were combined and redissolved with methanol, and its composition was determined with mass spectrometry.

#### 3.4.3. UPLC-QTOF/MS Analysis

A qualitative analyst was acquired using the UPLC-QTOF/MS analysis was conducted using the mass spectrometry ACQUITY UPLC I-Class System coupled to Xevo G2-S QTOF (Waters Corporation). The aqueous solution with 0.1% formic acid (*v*/*v*), methanol was treated as mobile phase A and B, respectively. Gradient elution was used starting with 5% B from 0 to 2.5 min, then increasing to 44% B until 26 min, with the addition in 60% B until 33 min, remaining at 100% B until 34 min. The flow rate was 0.3 mL/min, and the injection volume used was 3 μL. A reverse phase-column (Acquity UPLC^®^HSS T3, 1.7 μm; 2.1 × 100 mm) was used at 35 °C. The conditions for MS were set at ESI^+^ mode with a capillary voltage of 3 kV, the sample cone at 40 V, source temperature of 100 °C, acquisition range of 100–1500 D, and sampling rate of 20 points/sec. The collision energy ramp ranged 20–50 eV, and the MS collision energy was 10 eV.

### 3.5. The Lignan-Amides Preconcentration

The resulting residue was then subjected to HP-20 resin column and eluted successively by 40%, 60% EtOH. The 60% EtOH eluate was concentrated to give a brown group, and the sample was dissolved in methanol at 0.5 mg/mL and then stored at −20 °C after sealing for UPLC-MS/MS analysis for the treatment of LDS. Samples injected in UPLC/MS systems were filtered through a 0.22-µM membrane.

### 3.6. Cell Culture Assay

#### 3.6.1. Cell Culture

BV2 microglial cells were maintained in DMEM buffer mixed with 10% FBS and 1% penicillin/streptomycin. The cultivation conditions were 37 °C and 5% CO_2_.

#### 3.6.2. Cell Viability: CCK-8 Assay

A cell viability assay was performed according to the manufacturer’s instructions. Briefly, cells with a density of 5 × 10^6^ cells/well were plated in a 96-well plate and incubated overnight. The medium was moved, and the LDS (50–500 μg/mL) with indicated reagents was added. Controls with the fresh medium were included in the assay. The plates were incubated at 37 °C and 5% CO_2_ for 24 h. After the treatment, 10 μL of CCK-8 solution were added. The cells were incubated at 37 °C for 2 h. The absorbance of 450 nm was detected by a microplate reader. 

#### 3.6.3. LPS-Induced Model

DMEM (containing 10% FBS and 1% double-antibody) was used to dissolve 10 mg lipopolysaccharide (LPS) to prepare 10 mg/mL mother liquor, which was diluted to 1 μg/mL for modeling. In addition to the cytotoxicity test, the cells were pretreated with 1 μg/mL LPS for 12 h and aspirated. Then, 1 mM donepezil (DPZ) and LDS were administered for 12 h. The experiment was repeated three times.

#### 3.6.4. NO Release Analysis

Cells in the logarithmic growth phase were seeded in 96-well plates at a density of 5 × 10^6^ cells/well and cultured for 24 h before treatment. NaNO_2_ was used as the reference substance for preparing the standard curve of NO concentration calculation. Then, the NO concentration standard curve was used to calculate the NO release inhibition rate. The 1 μg/mL LPS was incubated in the cells for 12 h and then removed. According to the results of the cells viability test, the concentrations of LDS for NO release analysis were selected at 400, 200, and 100 μg/mL. The LDS were dissolved in the DMSO and then added into the fresh medium to get different concentrations. After 24 h culture, and NO detection kit determined the NO content according to the manufacturer’s instruction. DMSO was used as solvent control.

#### 3.6.5. Western Blotting

According to the results of the cells viability test and NO release analysis, the concentration of LDS for Western blotting was selected at 400, 200, and 100 μg/mL. After 24 h culture (LPS-incubated for 12 h and LDS-treated for 12 h), the cell proteins were extracted. The proteins were extracted in RIPA lysis buffer containing protease inhibitor cocktails. Samples containing 15 μg of protein were separated on SDS-PAGE gel and transferred onto nitrocellulose filter membrane (NC) membrane. After saturation with blocking buffer, the membranes were incubated with primary antibodies (iNOS, COX2, IBA-1, Tau, p-Tau, TREM2, DAP12, TLR4, MYD88, NLRP3, Caspase1, and GAPDH) at dilutions ranging from 1:1000 to 1:2000 at 4 °C overnight. After three washes with TBST, membranes were incubated with secondary antibodies. Signals were normalized to the GADPH signals to correct for unequal loading.

#### 3.6.6. Elisa Analysis

The supernatant samples of the cytokines were gained while the BV2 microglial cells were treated using the same procedure for Western blotting. The supernatant samples for IL-1β, IL-6, and TNFα cytokine levels were analyzed by the corresponding ELISA kits according to the instructions and were chosen at identical time points. The absorbance of each sample was read at 450 nm with a microplate spectrophotometer.

#### 3.6.7. Statistical Analysis

Triplicates of the measurements were expressed as mean ± standard error of the mean (SEM). All statistical analyses were performed using PRISM version 8.0 (GraphPad). Significant differences in differences were calculated using the Kruskal–Wallis test. Differences with a P value of less than 0.05 were considered statistically significant.

## 4. Conclusions

In conclusion, the SPR biosensor-UPLC/MS active ingredient recognition system of TREM2 was established, and the lignin-amides from the seed of *Datura metel* targeted TREM2 were discovered. In order to satisfy the further experimental study, 27 lignan-amides were preliminarily identified by UPLC-MS, which were compositions enriched by the HP-20 of *Datura metel* seeds. Moreover, the molecular mechanisms were investigated on BV2 cells induced by LPS between TREM2 and lignan-amides of *Datura metel* seeds. The results indicated that TREM2/DAP12 might mediate the down-regulation of TLR4/MyD88, NLRP3/Caspase1 signaling pathways, the AD signature protein of IBA-1/Tau, the inflammatory protein expression of iNOS/COX2, and the release of inflammatory factors TNF-α, IL-6, and IL-1β. On this basis, our study revealed the role of the lignan-amides in the treatment of inflammation-related AD from the *Datura metel* seeds. In addition, the DPZ was treated as a positive drug, which improved LPS-induced neuroinflammation of BV2 microglia by increasing TREM2/Dap12 protein expression and affecting TLR4/MyD88, NLRP3/caspase-1 pathway proteins. Thus, the expression of inflammatory proteins, including iNOS, COX2, and the release of inflammatory cytokines, such as TNF-α, IL-1β, and IL-6, were affected. The DPZ was used as a positive drug, and we chose it because of its better efficacy among known drugs [48]. It had previously been used to support the efficacy of the LDS and validate experimental methods. Interestingly, the LDS was found to be as effective as the DPZ in treating LPS-induced BV2 cell neuroinflammation.

According to the literature, the effects of LDS on TREM2 may be related to its inhibition of the production of inflammatory cytokines and the clearance of damaged cell fragments [49]. The TREM2 protein may also play a role by down-regulating CARD9 (caspase recruitment domain protein 9)-TRL4 and PI3K (phosphatidylinositol 3 kinase)-AKT (protein kinase B)-NF-κB (nuclear factor activated B cells κ-Light chain reinforcement) signaling pathways, which provided ideas for further research [50,51]. Meanwhile, TREM2 may affect the occurrence of CNS (central nervous system) diseases, such as AD, Parkinson’s disease (PD), Amyotrophic lateral sclerosis (ALS), stroke, as well as traumatic brain injury [52]. Notably, the LDS may also affect the occurrence of CNS diseases by regulating the expression of TREM2 protein. Therefore, the plant can be used as a prospective source of natural lignin-amides and can be developed as a potentially effective medicine or functional food. However, further studies should be performed on its in vivo pharmacological effects and related mechanisms.

## Figures and Tables

**Table 1:** Identification of TREM2-bound ingredients from *Datura metel* seeds.

Peak No.	Observed t_R_ (min)	Observed Ms (*m*/*z*)	Adduct	Compounds	Products Ions
1	12.80	625.2607	H	cannabisin G	625 [M+H]^+^ 488 [M+H-C_8_H_10_NO]^+^
2	12.94	643.2651	H	lyciumamide K	643 [M+H]^+^
3	13.87	625.2607	H	cannabisin F	625 [M+H]^+^ 462 [M+H–C_8_H_10_NO-CO]^+^
4	15.60	625.2607	H	cannabisin D	625 [M+H]^+^ 488 [M+H–C_8_H_10_NO]^+^ 462 [M+H–C_8_H_10_NO-CO]^+^

**Table 2:** Ion information of lignan-amides in LDS by mass spectrometry.

Peak No.	Observed t_R_ (min)	Observed MS (*m*/*z*)	Adduct	Compounds	Mass Error (ppm)	Products Ions	Reference
1	8.78	314.1388	H	N-trans-feruloyl tyramine	−1.3	314 [M+H]^+^ 177 [M+H C_8_H_10_NO]^+^	[18]
2	8.78	314.1388	H	N-cis-feruloyl tyramine	−1.3	314 [M+H]^+^ 177 [M+H-C_8_H_10_NO]^+^	[18]
3	8.80	510.2124	H	cannabisin H	2.4	510 [M+H]^+^ 312 [M+H-H_2_O-3CH_3_COOH]^+^	[18]
4	12.41	625.2547	H	cannabisin G (2)	−0.43	643 [M+H+H_2_O]^+^ 625 [M+H]^+^ 488 [M+H-C_8_H_10_NO]^+^	
5	12.50	643.2651	H	lyciumamide K	−0.67	643 [M+H]^+^ 625 [M+H-H_2_O]^+^ 462 [M+H-H_2_O-C_10_H_11_NO_2_]^+^	[18]
6	12.51	625.2550	H	Tribulusamide A	0.05	625 [M+H]^+^ 488 [M+H-C_8_H_10_NO]^+^ 462 [M+H-C_8_H_10_NO-H_2_O]^+^ 177 [M+H-C_27_H_26_NO_6_]^+^	[23]
7	12.75	492.2021	H	grossamide K	−0.21	492 [M+H]^+^ 462 [M+H-OCH_2_]^+^ 357 [M+H-C_8_H_10_NO]^+^	[18]
8	12.78	492.2021	H	cis-grossamide K	−0.21	492 [M+H]^+^ 462 [M+H-OCH_2_]^+^ 357 [M+H-C_8_H_10_NO]^+^	[15]
9	13.09	643.2657	H	cannabisin E	0.26	643 [M+H]^+^ 462 [M+H-H_2_O-C_9_H_11_NO]^+^ 113 [M+H-C_30_H_28_NO_8_]^+^	[16]
10	13.49	268.1342	H	N-trans-cinnamoyl tyramine	1.76	268 [M+H]^+^ 136 [M+H-C_7_H_16_O_3_]^+^ 128 [M+H-H_2_O-C_10_H_10_]^+^ 113 [M+H-C_11_H_7_O]^+^	[18]
11	13.89	625.2556	H	cannabisin F	1.01	625 [M+H]^+^	[18]
12	15.05	337.1552	H	N-trans-ferulyl tryptamine	0.02	337 [M+H]^+^ 177 [M+H C_10_H_11_N_2_]^+^	[24]
13	15.08	641.2493	H	compound 13 *	−0.9	641 [M+H]^+^	[31]
14	15.26	625.2553	H	cannabisin D	0.53	625 [M+H]^+^ 488 [M+H-C_8_H_10_NO]^+^ 462 [M+H-C_8_H_10_NO-CO]^+^	[18]
15	15.63	492.2021	H	lyciumamide C	−0.21	492 [M+H]^+^ 462 [M+H-OCH_3_]^+^	[26]
16	15.85	641.2493	H	Melongenamide B	−0.91	641 [M+H]^+^ 625 [M+H-H_2_O]^+^ 537 [M+H-3H_2_O-CH_3_COOH]^+^	[18]
17	16.45	657.2644	H+CH_3_COOH	cannabisin A	2.1	656 [M+H+CH_3_COOH]^+^ 494 [M+H+CH_3_COOH-C_9_H_10_NO_2_]^+^	[32]
18	17.06	671.2614	H	Melongenamide F	1.5	671 [M+H]^+^ 437 [M+H-C_12_H_13_NO_4_]^+^ 318 [M+H-C_20_H_21_NO_5_]^+^	[25]
19	17.35	625.2556	H	lyciumamide B	1.1	625 [M+H]^+^ 462 [M+H-H_2_O-CH_3_OH-C_8_H_11_NO]^+^	[18]
20	17.67	671.2471	H	Melongenamide G	1.5	671 [M+H]^+^ 639 [M+H-2H_2_O]^+^ 437 [M+2H-C_12_H_13_NO_4_]^+^ 419 [M+2H-C_12_H_13_NO_4_-H_2_O]^+^	[25]
21	17.86	625.2550	H+H_2_O	Chenoalbicin	−4.4	625 [M+H+H_2_O]^+^	[27]
22	18.86	625.2553	H	(2aS,3aS) lyciumamide D	0.37	625 [M+H]^+^ 113 [M+H-C_30_H_25_NO_7_]^+^	[18]
23	19.23	936.3699	H	Thoreliamide C	−0.45	936 [M+H]^+^ 625 [M+H-C_18_H_17_NO_4_]^+^ 454 [M+H-C_25_H_23_NO_9_]^+^ 113 [M+H-C_48_H_42_N_2_O_11_]^+^	[18]
24	20.82	623.2397	Na	lyciumamide F	4.3	623 [M+Na]^+^	[28]
25	22.17	625.2470	H	grossamide	−0.51	625 [M+H]^+^ 462 [M+H-C_9_H_9_NO_2_]^+^ 316 [M+H-C_18_H_18_NO_2_]^+^ 298 [M+H-H_2_O-C_18_H_18_NO_2_]^+^ 113 [M+H-C_30_H_25_NO_7_]^+^	[18]
26	22.53	936.3713	H	cannabisin O	0.6	936 [M+H]^+^ 599 [M+H-H_2_O-CH_3_OH-CO-C_17_H_17_NO_2_]^+^	[12]
27	23.94	538.2020	H+HCOOH	hibiscuwanin B	−0.37	538 [M+H+HCOOH]^+^ 439 [M+H+HCOOH-2H_2_O-2HOCH_3_]^+^	[18]

* Compound 13 was (1R)-N, N′-Bis(4-hydroxyphenethyl)-1*β*-(3,4-dihydroxyphenyl)-6,8-dimethoxy-7-hydroxy-1,2-dihydronaphthalene-2*α*, 3-dicarboxamide.

## Data Availability

Data is contained within the article or supplementary material. The data presented in this study are available in [insert article or supplementary material here].

## Supplementary Materials

The following are available online. Figures S1–S3: Mass spectrum of standard substance for peak 1–3 from the TREM2-binding, Figures S4–S30: Mass spectrums of peak 1–27 from the LDS.

## Sample Availability

Samples of the compounds are not available from the authors.

## Abbreviations

**Full Name**	**Acronyms**
Alzheimer’s disease	AD
triggering receptor expressed on myeloid Cells 2	TREM2
surface plasmon resonance	SPR
quadrupole time-of-flight tandem mass spectrometry	UPLC-MS
the lignin-amides from *Datura metel* seeds	LDS
inducible Nitric Oxide Synthase	iNOS
cyclooxygenase 2	COX-2
microtubule-associated protein tau	Tau
ionized calcium-binding adapter molecule 1	IBA-1
DNAX-activating protein of 12 kDa	DAP12
Toll-like receptor SX4	TLR4
Myeloid differentiation factor 88	MyD88
Recombinant NLR Family, Pyrin Domain Containing Protein 3	NLRP3
cysteinyl aspartate specific proteinase-1	Caspase-1
Inflammatory factors Interleukin 1 beta	IL-1β
Inflammatory factors Interleukin 6	IL-6
Tumor necrosis factor-alpha	TNFα
caspase recruitment domain protein 9	CARD9
phosphatidylinositol 3 kinase	PI3K
protein kinase B	AKT
nuclear factor activated B cells κ-Light chain reinforcement	NF-κB
central nervous system	CNS
Parkinson’s disease	PD
Amyotrophic lateral sclerosis	ALS

## References

[1] Wang S., Mustafa M., Yuede C.M., Salazar S.V., Kong P., Long H., Ward M., Siddiqui O., Paul R., Gilfillan S. (2020). Anti-human TREM2 induces microglia proliferation and reduces pathology in an Alzheimer’s disease model. J. Exp. Med..

[2] Lu Y., Liu W., Wang X. (2015). TREM2 variants and risk of Alzheimer’s disease: A meta-analysis. Neurol. Sci..

[3] Nicastro N., Malpetti M., Mak E., Williams G.B., O’Brien J.T. (2020). Grey matter changes related to microglial activation in alzheimer’s disease. Neurobiol. Aging.

[4] Konishi H., Kiyama H. (2020). Non-pathological roles of microglial trem2/DAP12: trem2/DAP12 regulates the physiological functions of microglia from development to aging. Neurochem. Int..

[5] Heslegrave A., Heywood W., Paterson R., Magdalinou N., Svensson J., Johansson P., Ohrfelt A., Blennow K., Hardy J., Schott J. (2016). Increased cerebrospinal fluid soluble TREM2 concentration in Alzheimer’s disease. Mol. Neurodegener..

[6] Ma L.Z., Tan L., Bi Y.L., Shen X.N., Xu W., Ma Y.H., Li H.Q., Dong Q., Yu J.T. (2020). Dynamic changes of CSF sTREM2 in preclinical Alzheimer’s disease: The CABLE study. Mol. Neurodegener..

[7] Wang Y.H., Yang H., Cheng X., Yang Y.L., Lyu Y., Du G.H. (2017). P7 extract of ligusticum chuanxiong hort attenuates lps-induced neuroinflammation in bv2 microglia via down-regulation of tlr4/myd88 pathway. Biochem. Pharm..

[8] Wang Y., Cella M., Mallinson K., Ulrich J.D., Young K.L., Robinette M.L., Gilfillan S., Krishnan G.M., Sudhakar S., Zinselmeyer B.H. (2015). TREM2 lipid sensing sustains the microglial response in an Alzheimer’s disease model. Cell.

[9] Cui X., Qiao J., Liu S., Wu M., Gu W. (2021). Mechanism of trem2/DAP12 complex affecting β-amyloid plaque deposition in alzheimer’s disease modeled mice through mediating inflammatory response. Brain Res. Bull..

[10] Ewers M., Franzmeier N., Suárez-Calvet M., Morenas-Rodriguez E., Caballero M.A.A., Kleinberger G., Piccio L., Cruchaga C., Deming Y., Dichgans M. (2019). Increased soluble TREM2 in cerebrospinal fluid is associated with reduced cognitive and clinical decline in Alzheimer’s disease. Sci. Transl. Med..

[11] Tanaka M., Saito S., Inoue T., Satoh-Asahara N., Ihara M. (2020). Potential Therapeutic Approaches for Cerebral Amyloid Angiopathy and Alzheimer’s Disease. Int. J. Mol. Sci..

[12] Yan X., Tang J., Passos C.D.S., Nurisso A., Simoes-Pires C., Ji M., Lou H., Fan P. (2015). Characterization of Lignanamides from Hemp (*Cannabis. sativa.* L.) Seed and their Antioxidant and Acetylcholinesterase Inhibitory Activities. J. Agric. Food Chem..

[13] Feng X., Li H., Gao H., Huang Y., Zhou W., Yu Y., Yao X. (2016). Bioactive Nitrogenous Compounds from Acorus tatarinowii. Magn. Reson. Chem..

[14] Wang S., Luo Q., Fan P. (2019). Cannabisin F from hemp (*Cannabis sativa.*) seed suppresses lipopolysaccharide-induced inflammatory responses in bv2 microglia as sirt1 modulator. Int. J. Mol. Sci..

[15] Zhou Y., Wang S., Ji J., Lou H., Fan P. (2018). Hemp (*Cannabis sativa.* L.) seed phenylpropionamides composition and effects on memory dysfunction and biomarkers of neuroinflammation induced by lipopolysaccharide in mice. ACS Omega.

[16] Nigro E., Crescente G., Formato M., Pecoraro M.T., Pacifico S. (2020). Hempseed lignanamides rich-fraction: Chemical investigation and cytotoxicity towards u-87 glioblastoma cells. Molecules.

[17] Bingyou Y., Haibing J., Yan L., Zhenpeng X., Haixue K. (2018). Study on the chemical constituents of the seeds of *Datura. Metel.* (IV). Chin. Med. Mater..

[18] Bingyou Y., Yan L., Xin W., Yonggang X., Qiuhong W., Haixue K. (2013). Studies on the chemical constituents of the seeds of *Datura. metel.* (I). Chin. Herb. Med..

[19] Bingyou Y., Yan L., Peiyan Z., Qiuhong W., Haixue K. (2015). Research on the chemical constituents of alkaloids in the seeds of *Datura. metel*. Proceedings of the World Conference on Traditional Chinese Medicine Summer Summit and “One Belt One Road” International Symposium on the Development of Traditional Chinese Medicine.

[20] You Y.B., Xue K.H., Hui Y.S., Yan L., Ping S.Y. (2020). Identification and Quantification of Alkaloid Compounds from Different Parts and Production Areas of *Datura metel.* L.. Heterocycles.

[21] Murthy B.K., Nammi S., Kota M.K., Rao R., Rao N.K., Annapurna A. (2004). Evaluation of hypoglycemic and antihyperglycemic effects of *Datura metel.* (linn.) seeds in normal and alloxan-induced diabetic rats. J. Ethnopharmacol..

[22] Wannang N.N., Ndukwe H.C., Nnabuife C. (2009). Evaluation of the analgesic properties of the *Datura metel.* seeds aqueous extract. J. Med. Plants Res..

[23] Bing-You Y., Hai-Bing J., Yan L., Jing C., Hai-Xue K. (2020). Steroids from the seeds of *Datura metel*. J. Asian Nat. Prod. Res..

[24] Handrick V., Vogt T., Frolov A. (2010). Profiling of hydroxycinnamic acid amides in Arabidopsis thaliana pollen by tandem mass spectrometry. Anal. Bioanal. Chem..

[25] Li J.-X., Shi Q., Xiong Q.-B., Prasain J., Tezuka Y., Hareyama T., Wang Z.-T., Tanaka K., Namba T., Kadota S. (1998). Tribulusamide A and B, new hepatoprotective lignan amides from the fruits of *Tribulus terrestris*: Indications of cytoprotective activity in murine hepatocyte culture. Planta Med..

[26] Young S.J., Ae K.M., Jo K.M., Wanjoo C., Yongsoo K. (2014). Acetylcholinesterase Inhibitors from the Stem of Zea mays. Nat. Prod. Sci..

[27] Yang B.Y., Yin X., Liu Y., Ye H.L., Kuang H.X. (2019). Bioassay-guided isolation of lignan amides with potential anti-inflammatory effect from the roots of *Solanum melongena.* L.. Phytochem. Lett..

[28] Zhu P.F., Dai Z., Wang B., Wei X., Yu H.F., Yan Z.R., Zhao X., Liu Y., Luo X. (2017). The anticancer activities phenolic amides from the stem of *Lycium barbarum*. Nat. Prod. Bioprospect..

[29] Cutillo F., D’Abrosca B., Dellagreca M., Zarrelli A. (2010). Chenoalbicin, a Novel Cinnamic Acid Amide Alkaloid from *Chenopodium album*. Chem. Bio..

[30] Chen H., Li Y.J., Sun Y.J., Gong J.-H., Du K., Zhang Y.-L., Su C.-F., Han Q.-Q., Zheng X.-K., Feng W.S. (2017). Lignanamides with potent antihyperlipidemic activities from the root bark of *Lycium chinense*. Fitoterapia.

[31] National Center for Biotechnology Information (2021). PubChem Compound Summary for CID 101938451. https://pubchem.ncbi.nlm.nih.gov/compound/101938451.

[32] Choi H.S., Cho J.-Y., Jin M.R., Lee Y.G., Kim S.-J., Ham K.-S., Moon J.-H. (2016). Phenolics, acyl galactopyranosyl glycerol, and lignan amides from *Tetragonia tetragonioides.* (Pall.) Kuntze. Food Sci. Biotechnol..

[33] Luo Q., Yan X., Bobrovskaya L., Ji M., Yuan H., Lou H., Fan P. (2017). Anti-neuroinflammatory effects of grossamide from hemp seed via suppression of TLR-4-mediated NF-κB signaling pathways in lipopolysaccharide-stimulated BV2 microglia cells. Mol. Cell Biochem..

[34] Zhou Y., Wang S., Lou H., Fan P. (2018). Chemical constituents of hemp (*Cannabis sativa.* L.) seed with potential anti-neuroinflammatory activity. Phytochem. Lett..

[35] Wang Y., Ji S., Zang W., Wang N., Cao J., Li X., Sun C. (2019). Identification of phenolic compounds from a unique citrus species, finger lime (citrus australasica) and their inhibition of lps-induced no-releasing in bv-2 cell line. Food Chem. Toxicol..

[36] Wang Y.H., Lv H.N., Cui Q.H., Tu P.F., Zeng K.W. (2020). Isosibiricin inhibits microglial activation by targeting the dopamine d1/d2 receptor-dependent nlrp3/caspase-1 inflammasome pathway. Acta Pharm. Sin..

[37] Sloane J.A., Hollander W., Moss M.B., Rosene D.L., Carmela R. (1999). Abraham Increased microglial activation and protein nitration in white matter of the aging monkey. Neurobiol. Aging.

[38] Wysscoray T., Lucin K.M. (2009). Immune activation in brain aging and neurodegeneration: Too much or too little. Neuron.

[39] El Khoury J., Toft M., Hickman S.E., Means T.K., Terada K., Geula C., Luster A.D. (2007). Ccr2 Deficiency Impairs Microglial Accumulation and Accelerates Progression of Alzheimer’s Disease. Nat. Med..

[40] Kitazawa M., Oddo S., Yamasaki T.R., Green K.N., LaFerla F.M. (2005). Lipopolysaccharide-Induced Inflammation Exacerbates Tau Pathology by a Cyclin-Dependent Kinase 5-Mediated Pathway in a Transgenic Model of Alzheimer’s Disease. J. Neurosci..

[41] Lee D.C., Rizer J., Selenica M.-L.B., Reid P., Kraft C., Johnson A., Blair L., Gordon M.N., Dickey C., Morgan D. (2010). LPS-induced inflammation exacerbates phospho-tau pathology in rTg4510 mice. J. Neuroinflamm..

[42] Papasozomenos S.C., Binder L.I. (1987). Phosphorylation determines two distinct species of tau in the central nervous system. Cell. Motil. Cytoskelet..

[43] Ghoshal N., García-Sierra Fu Y., Beckett L.A., Mufson E.J., Kuret J., Berry R.W., Binder L.I. (2010). Tau-66: Evidence for a novel tau conformation in Alzheimer’s disease. J. Neurochem..

[44] Uchihara T., Tsuchiya K., Nakamura A., Ikeda K. (2000). Appearance of tau-2 immunoreactivity in glial cells in human brain with cerebral infarction. Neurosci. Lett..

[45] Uchihara T., Nakamura A., Arai T., Ikeda K., Tsuchiya K. (2004). Microglial tau undergoes phosphorylation-independent modification after ischemia. Glia.

[46] Majerova P., Zilkova M., Kazmerova Z., Kovac A., Paholikova K., Kovacech B., Zilka N., Novak M. (2014). Microglia display modest phagocytic capacity for extracellular tau oligomers. J. Neuroinflamm..

[47] Singh A., Mishra G., Maurya A., Rajendra A., Komal K., Abhimanyu T., Arati R., Gopal R.K., Bhupesh S., Giriraj K.T. (2019). Role of TREM2 in Alzheimer’s disease and its consequences on β-amyloid, Tau and neurofibrillary tangles. Curr. Alzheimer Res..

[48] Marucci G., Moruzzi M., Amenta F. (2020). Donepezil in the treatment of Alzheimer’s disease. Diagnosis and Management in Dementia.

[49] Atagi Y., Liu C., Painter M.M., Chen X., Verbeeck C., Zheng H., Li X., Rademakers R., Kang S.S., Xu H. (2015). Apolipoprotein E Is a Ligand for Triggering Receptor Expressed on Myeloid Cells 2 (TREM2). J. Biol. Chem..

[50] Qin Z., Gu M., Zhou J., Zhang W., Zhao N., Lü Y., Yu W. (2020). Triggering receptor expressed on myeloid Cells 2 activation downregulates toll-like receptor 4 expression and ameliorates cognitive impairment in the Aβ1-42-induced Alzheimer’s disease mouse model. Synapse.

[51] Li C., Zhao B., Lin C., Gong Z., An X. (2019). TREM2 inhibits inflammatory responses in mouse microglia by suppressing the PI3K/NF-κB signaling. Cell Biol. Int..

[52] Gratuze M., Leyns C., Holtzman D.M. (2018). New insights into the role of TREM2 in Alzheimer’s disease. Mol. Neurodegener..

