# Supplementary material for: Discovery of Active Ingredients Targeted TREM2 by SPR Biosensor-UPLC/MS Recognition System, and Investigating the Mechanism of Anti-Neuroinflammatory Activity on the Lignin-Amides from Datura metel Seeds
Source: Molecules. 2021 Sep 30;26(19):5946. doi: 10.3390/molecules26195946 (PMC8512677; doi:10.3390/molecules26195946)
Supplement: Supplementary file 1 [file molecules-26-05946-s001.zip › molecules-1385009-supplementary.pdf]

Supporting Information

Article

Discovery of Active Ingredients Targeted TREM2 by SPR Biosensor-UPLC/MS Recognition System, and Investigating the Mechanism of Anti-neuroinflammatory Activity on the Lignin-amides from *Datura metel* Seeds

Si-Yi Wang †, Yan Liu †, Xiao-Mao Li, Adnan Mohammed Algradi, Hai Jiang, Yan-Ping Sun, Wei Guan, Juan Pan, Hai-Xue Kuang* and Bing-You Yang*

Key Laboratory of Chinese Materia, Heilongjiang University of Chinese Medicine, Ministry of Education, Harbin 150040, China; WSY06182021@163.com (S.Y.W.); lifeliuyan@163.com (Y.L.); 18216074841@163.com (X.M.L), dradnan86@hotmail.com (A.M.A); JiangHai_777@126.com (H.J.); 18704608056@163.com (Y.P.S.); myguanwei1234@yeah.net (W.G.); panjuan1002@hotmail.com (J.P)
* Correspondence: yangbingyou@hljucm.net (B.Y.Y.); hxkuang@yahoo.com (H.X.K.)

† These authors contributed equally to this work

Contents:

Figure S1. Mass spectrum of standard substance for peak 1 from the TREM2-binding
Figure S2. Mass spectrum of standard substance for peak 3 from theTREM2-binding
Figure S3. Mass spectrum of standard substance for peak 4 from the TREM2-binding
Figure S4. Mass spectrum of peak 1 from the LDS
Figure S5. Mass spectrum of peak 2 from the LDS
Figure S6. Mass spectrum of peak 3 from the LDS
Figure S7. Mass spectrum of peak 4 from the LDS
Figure S8. Mass spectrum of peak 5 from the LDS
Figure S9. Mass spectrum of peak 6 from the LDS
Figure S10. Mass spectrum of peak 7 from the LDS
Figure S11. Mass spectrum of peak 8 from the LDS
Figure S12. Mass spectrum of peak 9 from the LDS
Figure S13. Mass spectrum of peak 10 from the LDS
Figure S14. Mass spectrum of peak 11 from the LDS
Figure S15. Mass spectrum of peak 12 from the LDS
Figure S16. Mass spectrum of peak 13 from the LDS
Figure S17. Mass spectrum of peak 14 from the LDS
Figure S18. Mass spectrum of peak 15 from the LDS
Figure S19. Mass spectrum of peak 16 from the LDS
Figure S20. Mass spectrum of peak 17 from the LDS
Figure S21. Mass spectrum of peak 18 from the LDS
Figure S22. Mass spectrum of peak 19 from the LDS
Figure S23. Mass spectrum of peak 20 from the LDS
Figure S24. Mass spectrum of peak 21 from the LDS
Figure S25. Mass spectrum of peak 22 from the LDS
Figure S26. Mass spectrum of peak 23 from the LDS
Figure S27. Mass spectrum of peak 24 from the LDS
Figure S28. Mass spectrum of peak 25 from the LDS
Figure S29. Mass spectrum of peak 26 from the LDS
Figure S30. Mass spectrum of peak 27 from the LDS
Figure S31. Kruskal-Wallis analysis results for each group
Table S1. The results for TREM2 of Kruskal-Wallis
Table S2. The results for Dap12 of Kruskal-Wallis
Table S3. The results for iNOS of Kruskal-Wallis
Table S4. The results for COX-2 of Kruskal-Wallis
Table S5. The results for TLR4 of Kruskal-Wallis
Table S6. The results for MyD88 of Kruskal-Wallis
Table S7. The results for NLRP3 of Kruskal-Wallis
Table S8. The results for Caspase-1 of Kruskal-Wallis
Table S9. The results for IBA-1 of Kruskal-Wallis

Citation: Wang, S.-Y.; Liu, Y.; Li, X.-M.; Algradi, A.M.; Jiang, H.; Sun, Y.-p.; Guan, W.; Pan, J.; Kuang, H.-X.; Yang, B.-Y.; et al. Discovery of active ingredients targeted TREM2 by SPR biosensor-UPLC/MS recognition system, and investigating the mechanism of anti-neuroinflammatory activity on the lignin-amides from *Datura metel* seeds. 2021, 24, x. https://doi.org/10.3390/xxxxx

Academic editor：Paola Barraja

Received: 1 September 2021
Accepted: 27 September 2021
Published: date

Publisher's Note: MDPI stays neutral with regard to jurisdictional claims in published maps and institutional affiliations.

Copyright: © 2021 by the authors. Submitted for possible open access publication under the terms and conditions of the Creative Commons Attribution (CC BY) license (http://creativecommons.org/licenses/by/4.0/).

Table S10. The results for p-Tau/Tau of Kruskal-Wallis

Figure S1. Mass spectrum of standard substance for peak 1 from the TREM2-binding

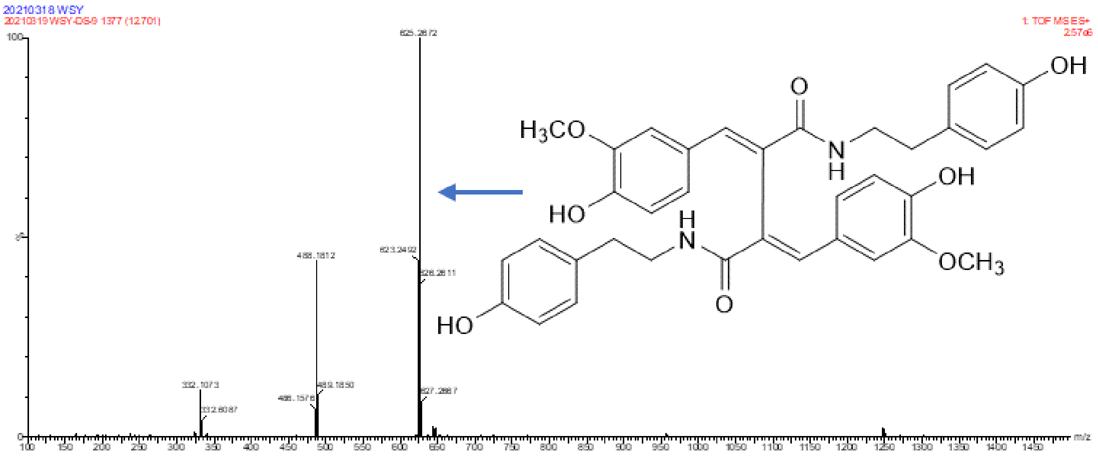

Figure S2. Mass spectrum of standard substance for peak 3 from the TREM2-binding

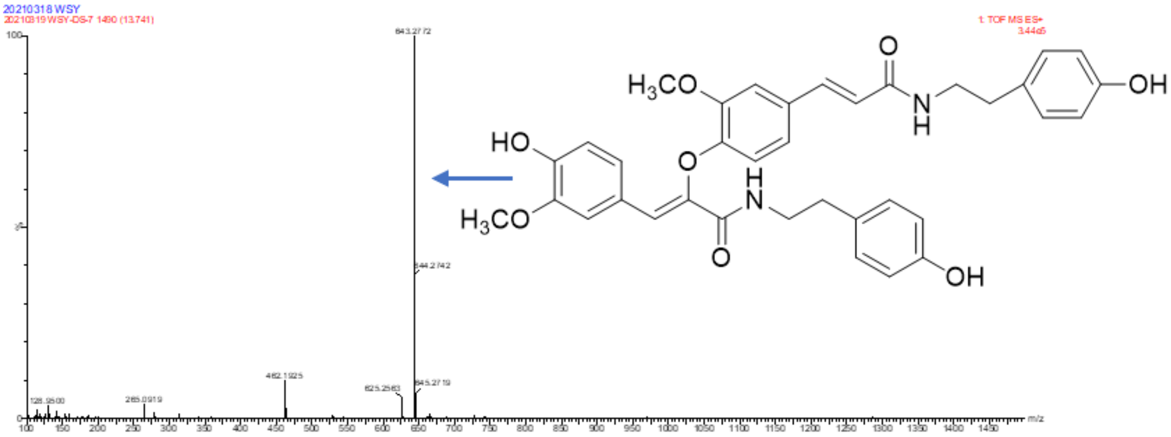

Figure S3. Mass spectrum of standard substance for peak 4 from the TREM2-binding

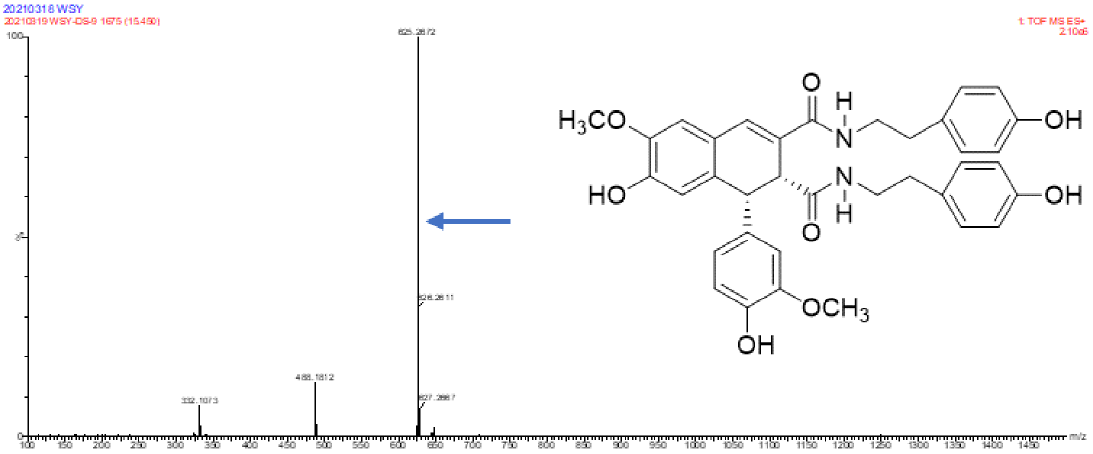

Figure S4. Mass spectrums of peak 1 from the LDS

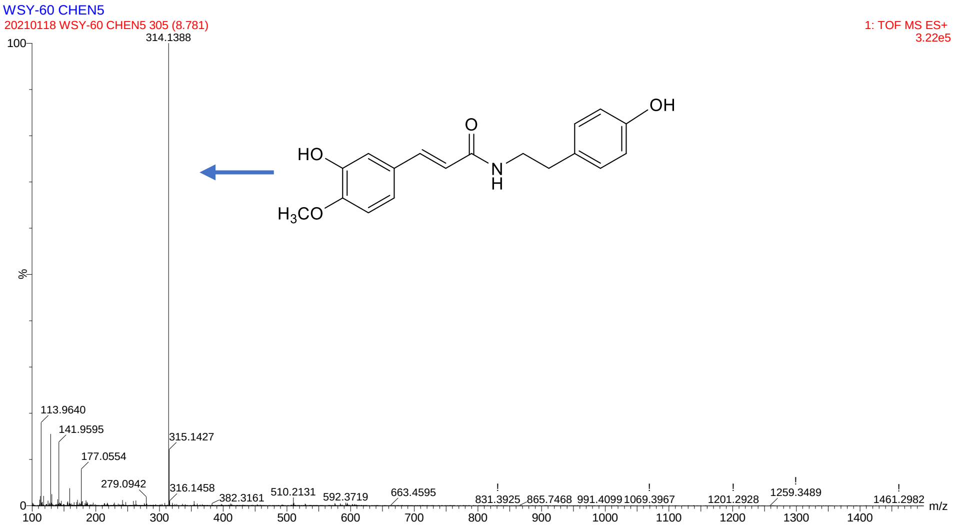

Figure S5. Mass spectrum of peak 2 from the LDS

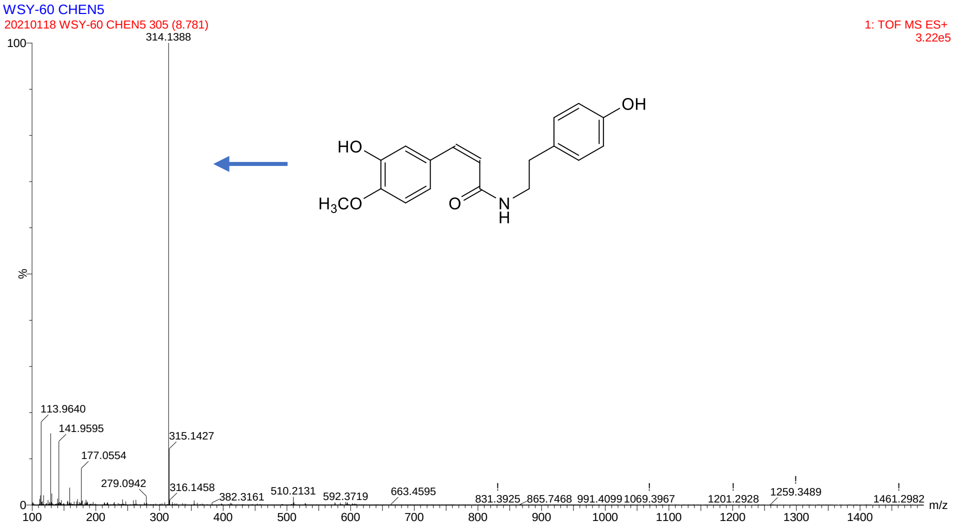

Figure S6. Mass spectrum of peak 3 from the LDS

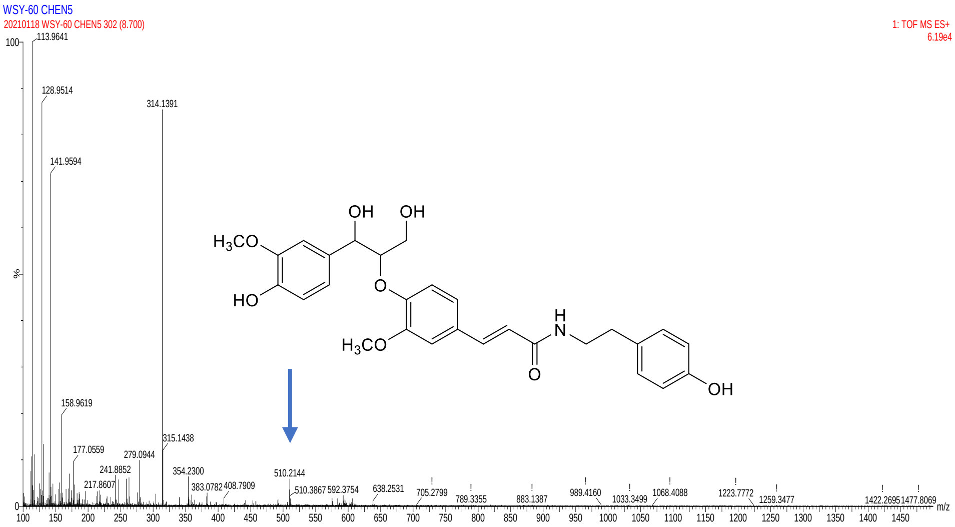

Figure S7. Mass spectrum of peak 4 from the LDS

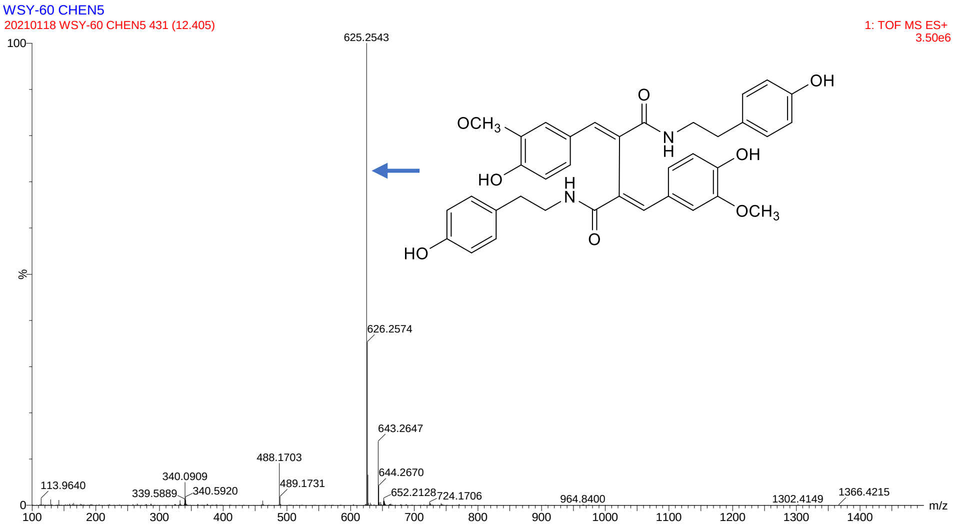

Figure S8. Mass spectrum of peak 5 from the LDS

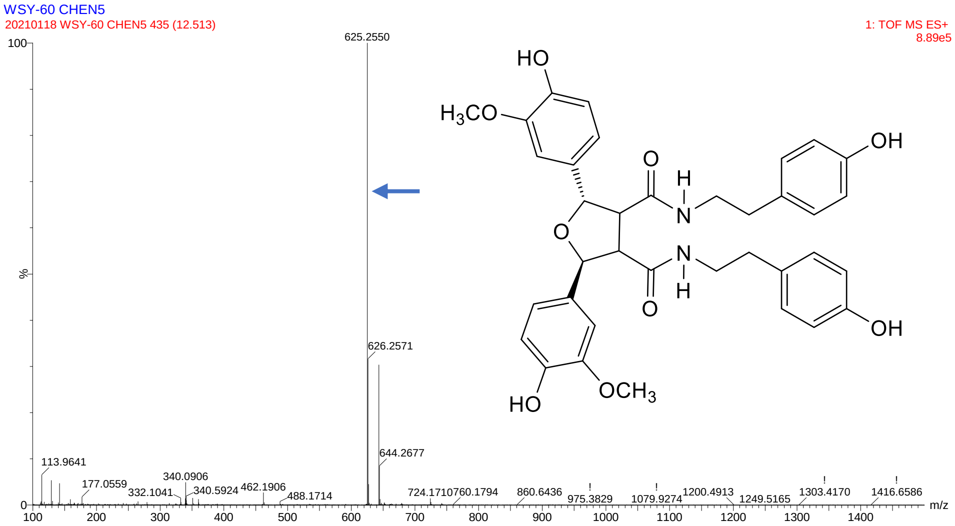

Figure S9. Mass spectrum of peak 6 from the LDS

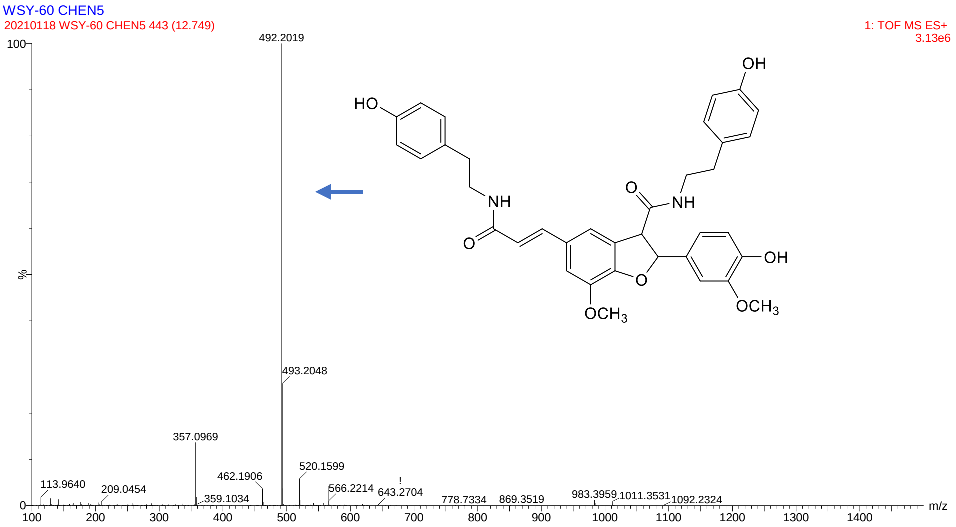

Figure S10. Mass spectrum of peak 7 from the LDS

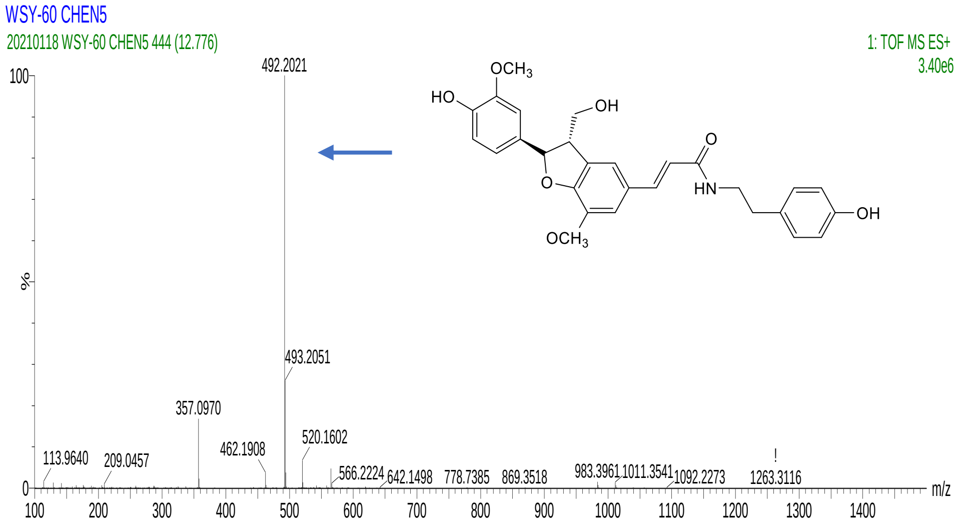

Figure S11. Mass spectrum of peak 8 from the LDS

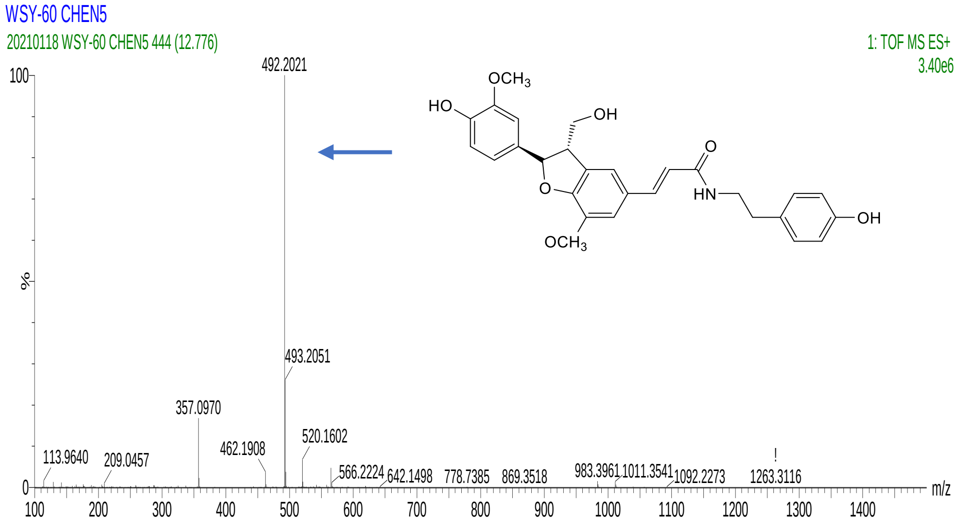

Figure S12. Mass spectrum of peak 9 from the LDS

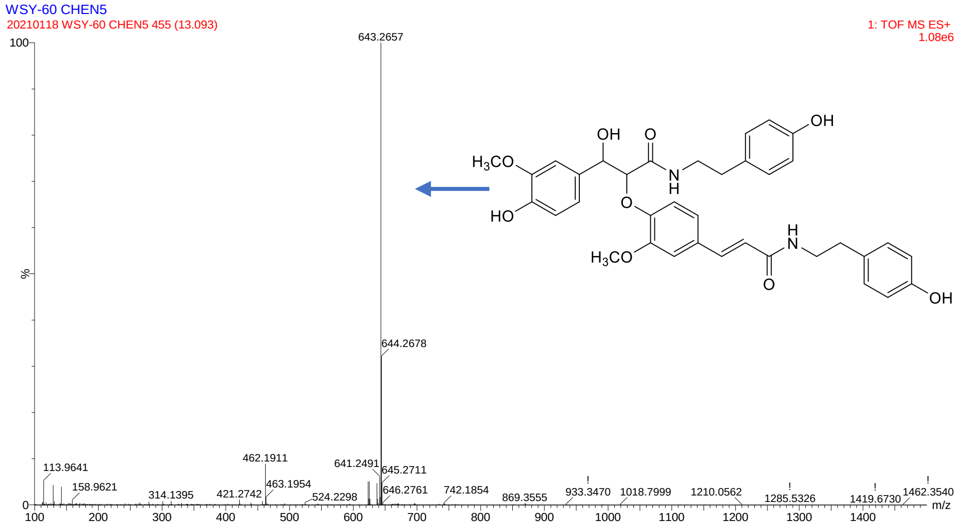

Figure S13. Mass spectrum of peak 10 from the LDS

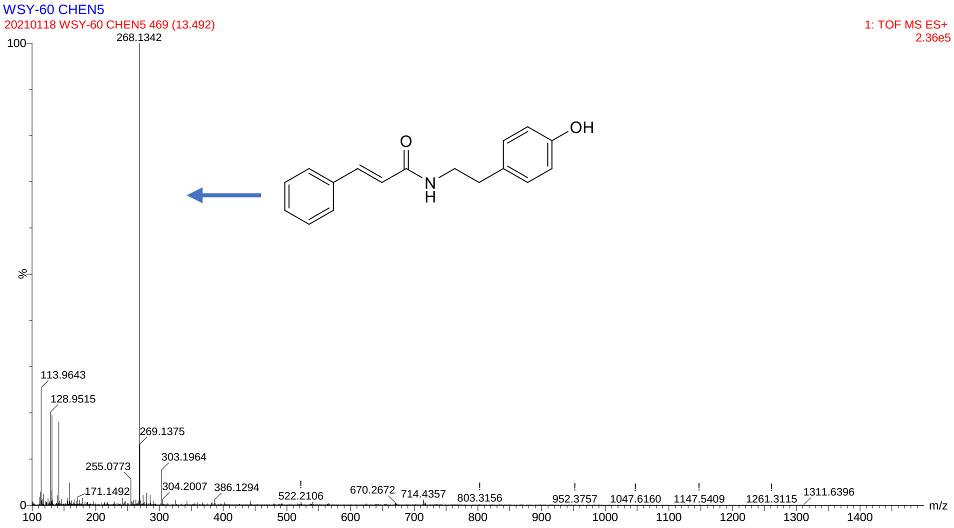

Figure S14. Mass spectrum of peak 11 from the LDS

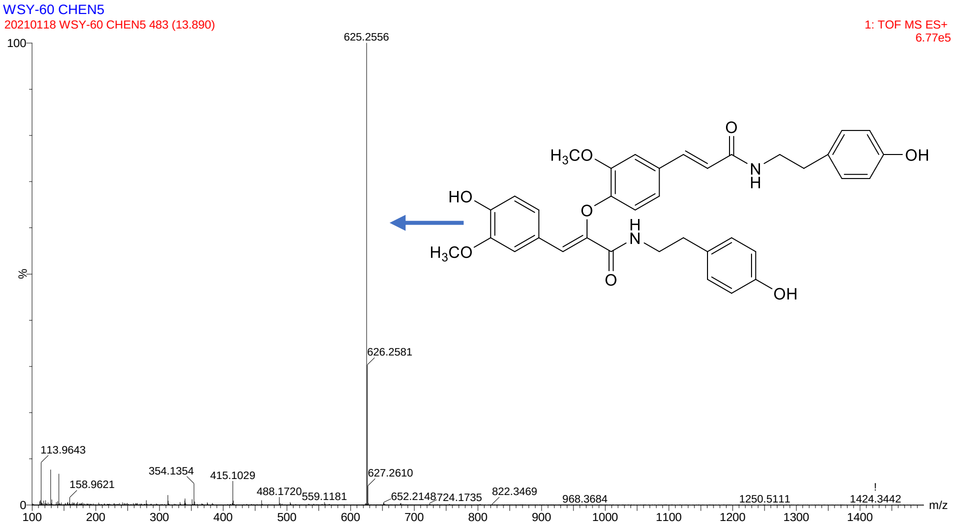

Figure S15. Mass spectrum of peak 12 from the LDS

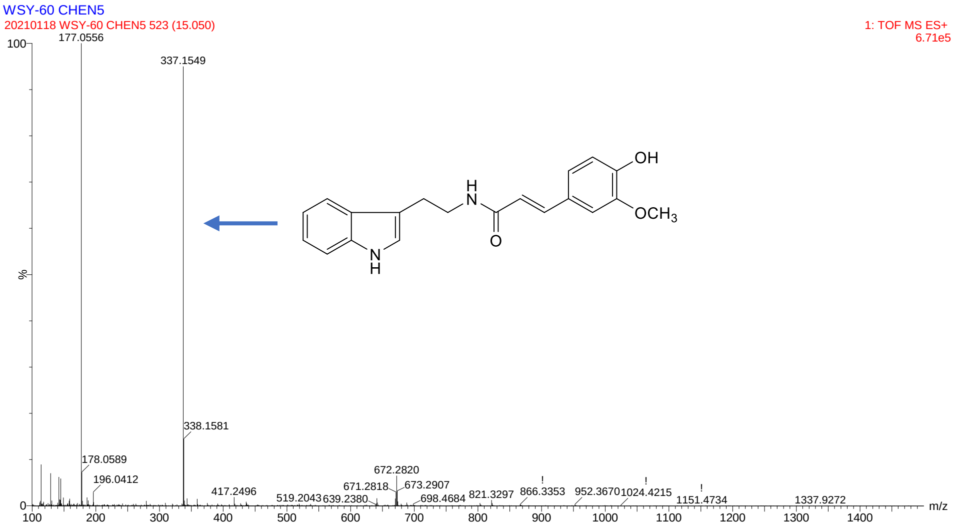

Figure S16. Mass spectrum of peak 13 from the LDS

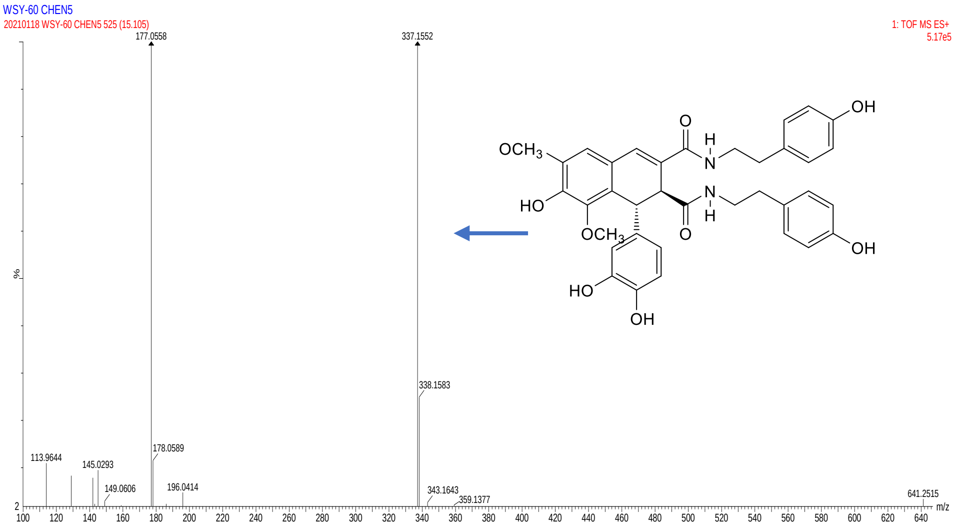

Figure S17. Mass spectrum of peak 14 from the LDS

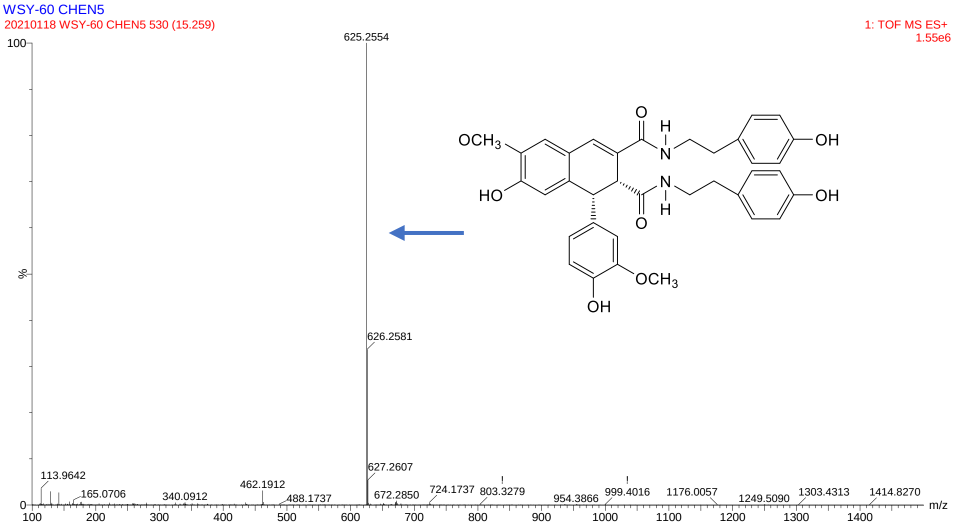

Figure S18. Mass spectrum of peak 15 from the LDS

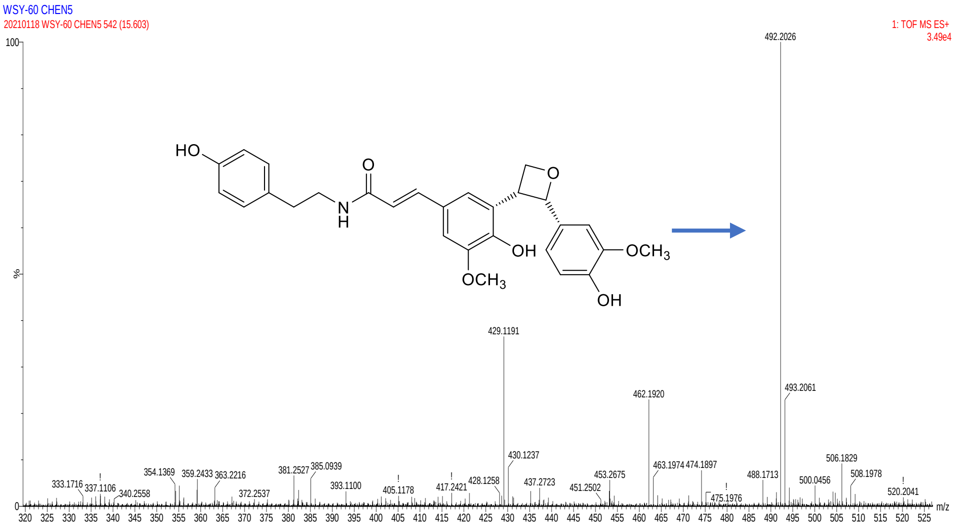

Figure S19. Mass spectrum of peak 16 from the LDS

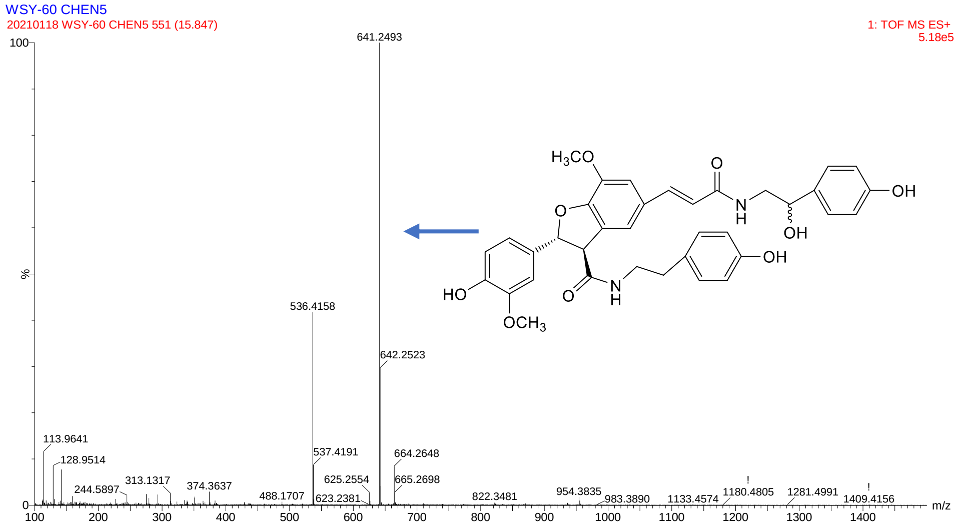

Figure S20. Mass spectrum of peak 17 from the LDS

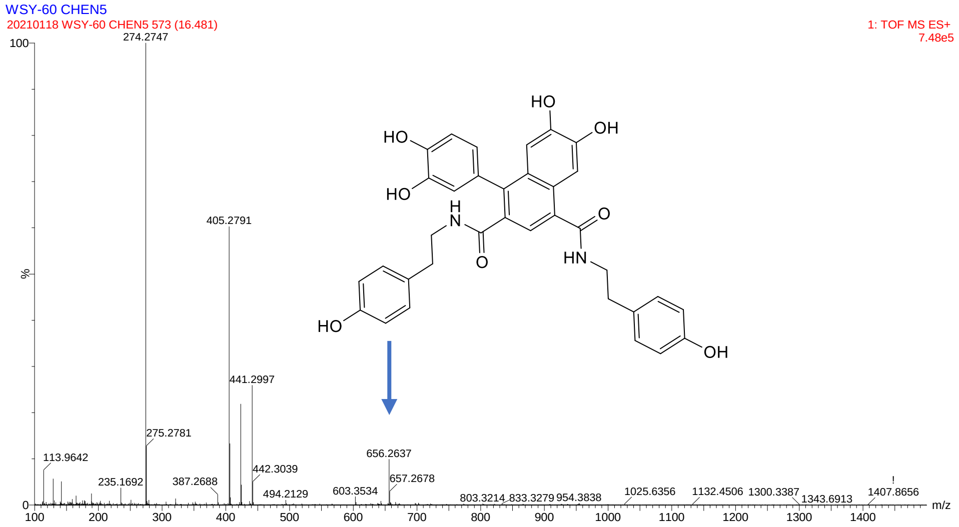

Figure S21. Mass spectrum of peak 18 from the LDS

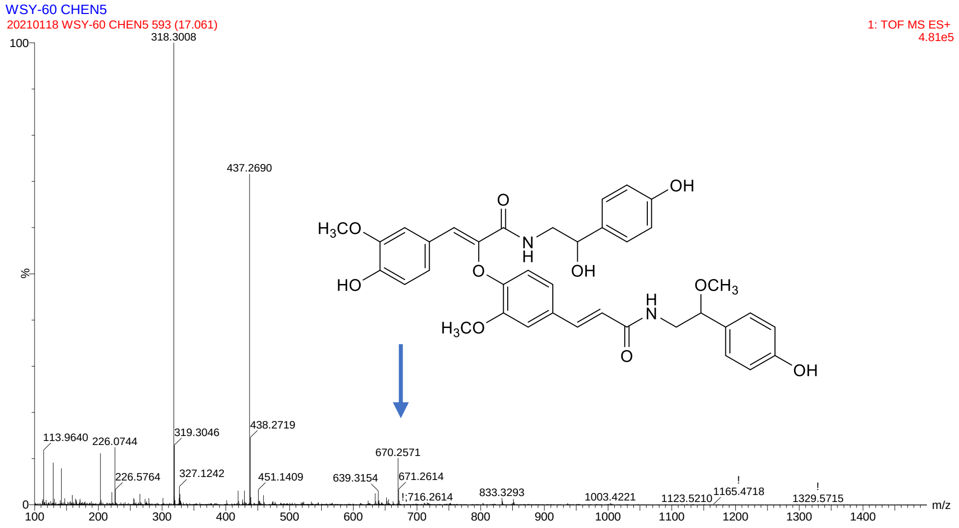

Figure S22. Mass spectrum of peak 19 from the LDS

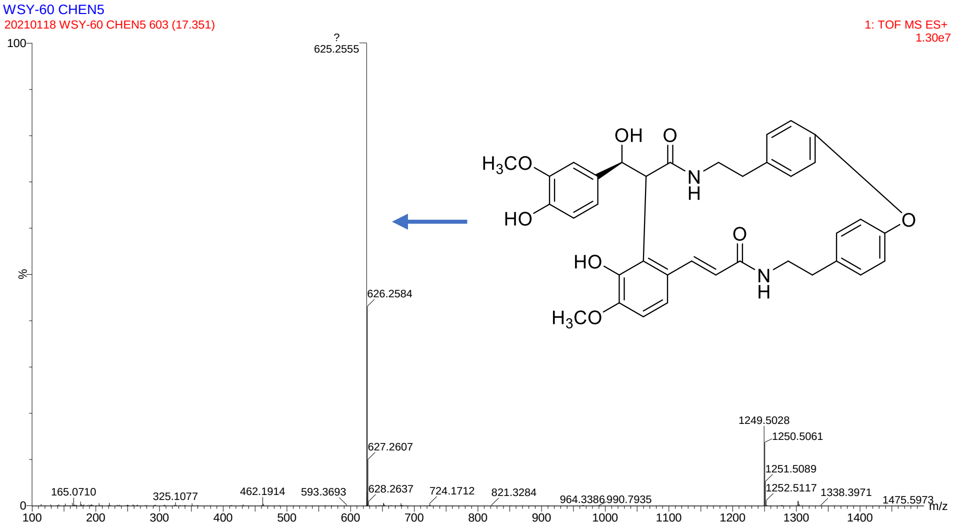

Figure S23. Mass spectrum of peak 20 from the LDS

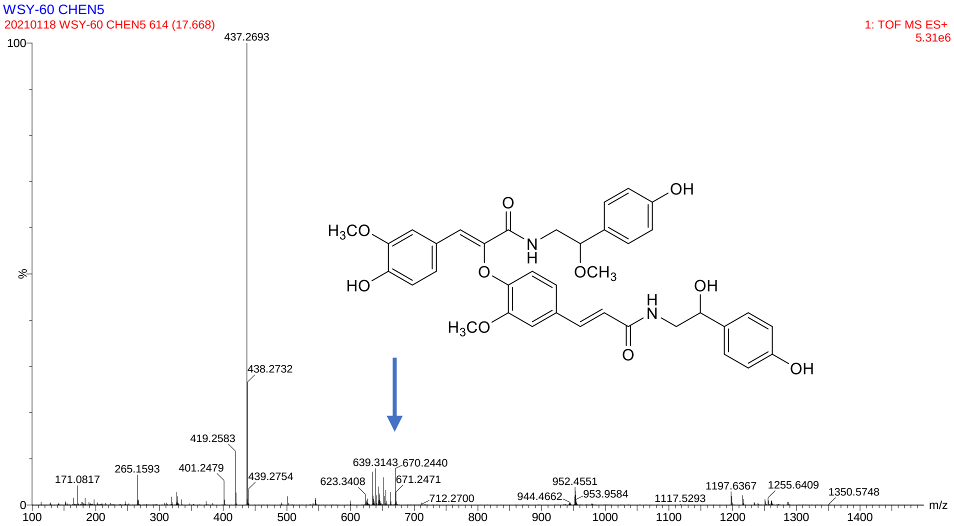

Figure S24. Mass spectrum of peak 21 from the LDS

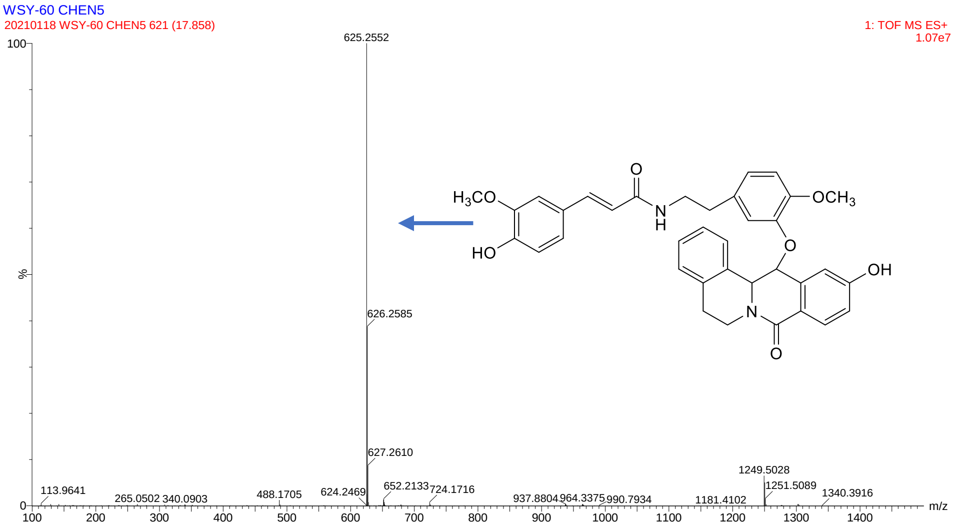

Figure S25. Mass spectrum of peak 22 from the LDS

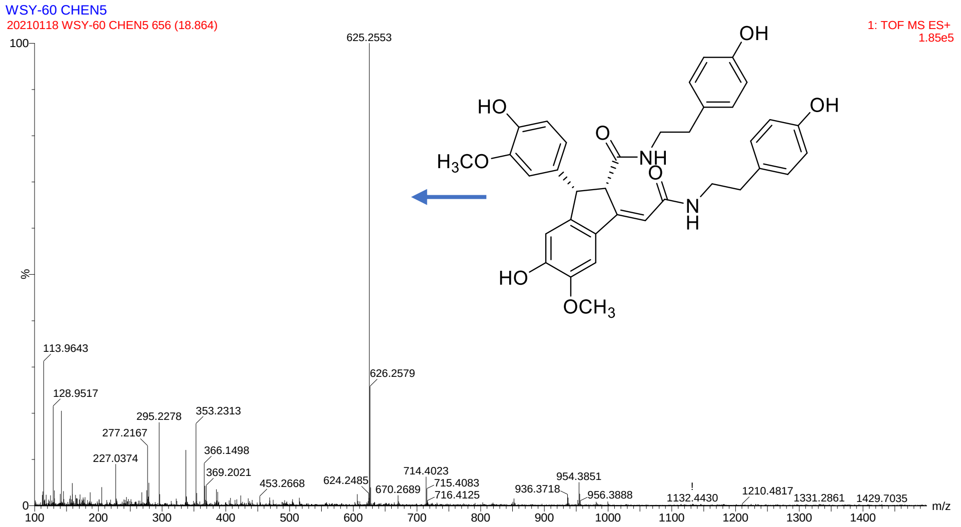

Figure S26. Mass spectrum of peak 23 from the LDS

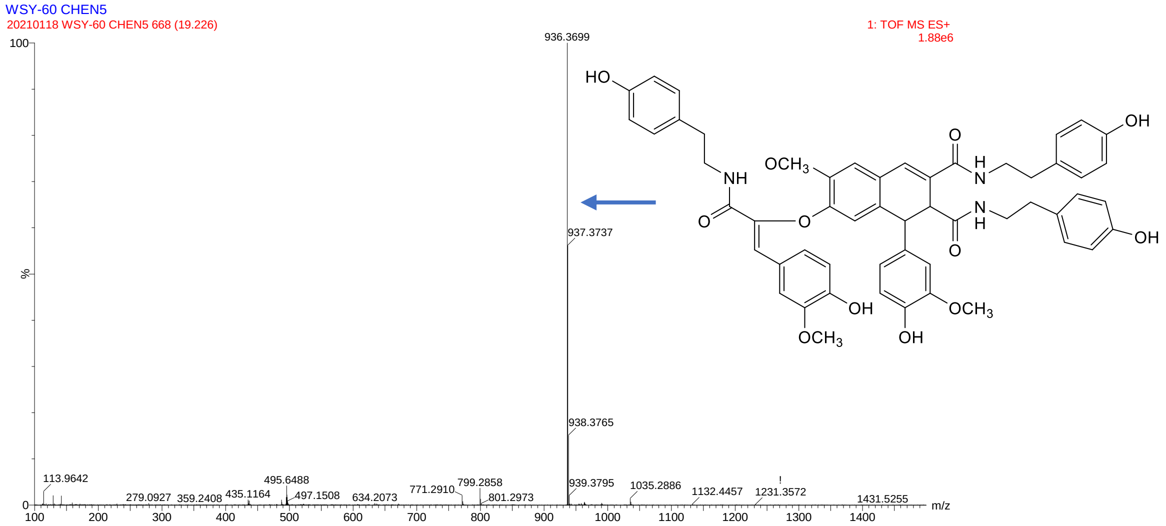

Figure S27. Mass spectrum of peak 24 from the LDS

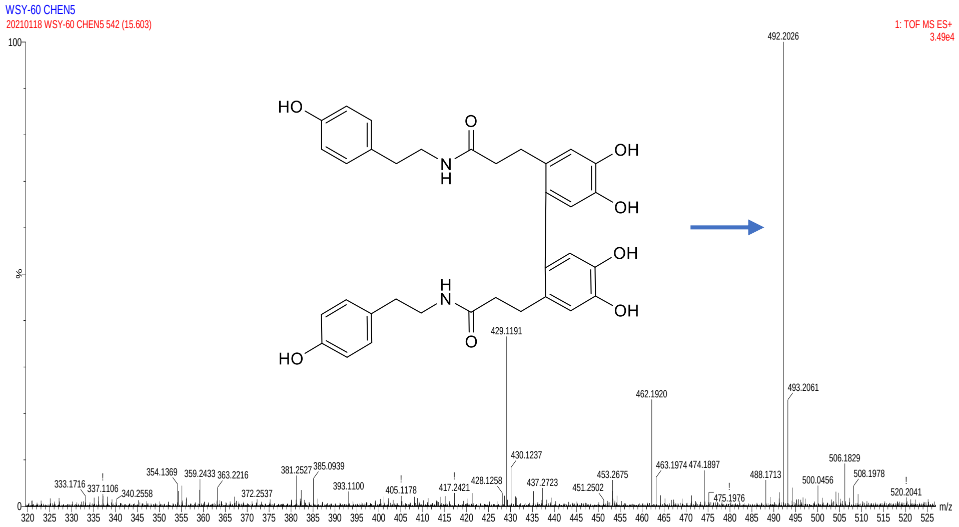

Figure S28. Mass spectrum of peak 25 from the LDS

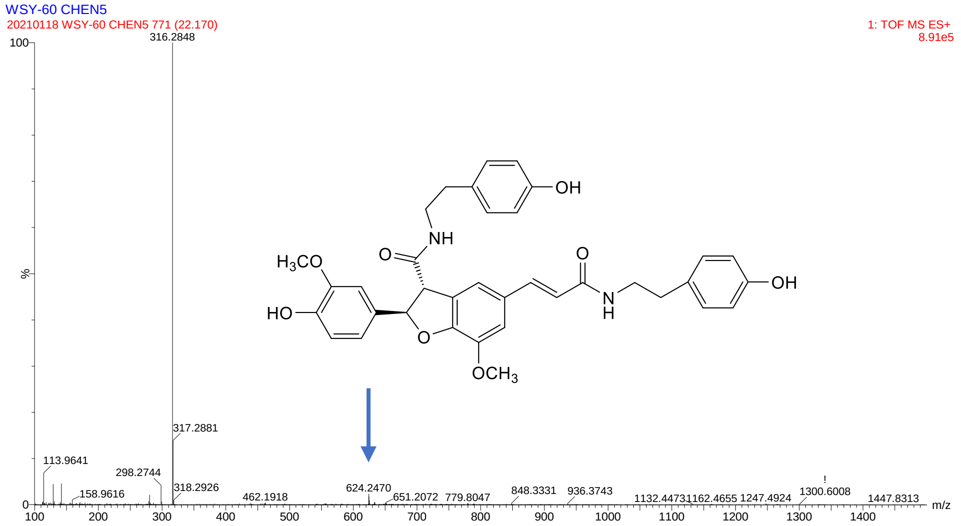

Figure S29. Mass spectrum of peak 26 from the LDS

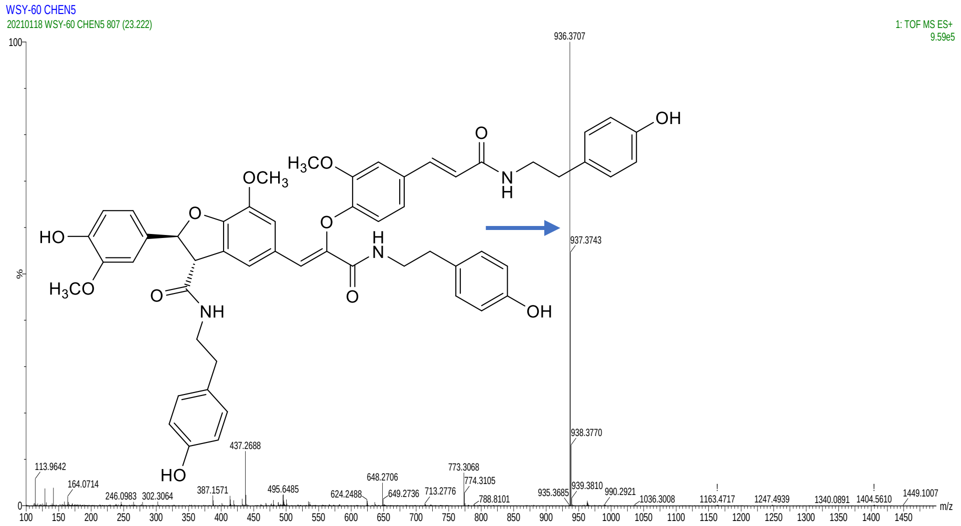

Figure S30. Mass spectrum of peak 27 from the LDS

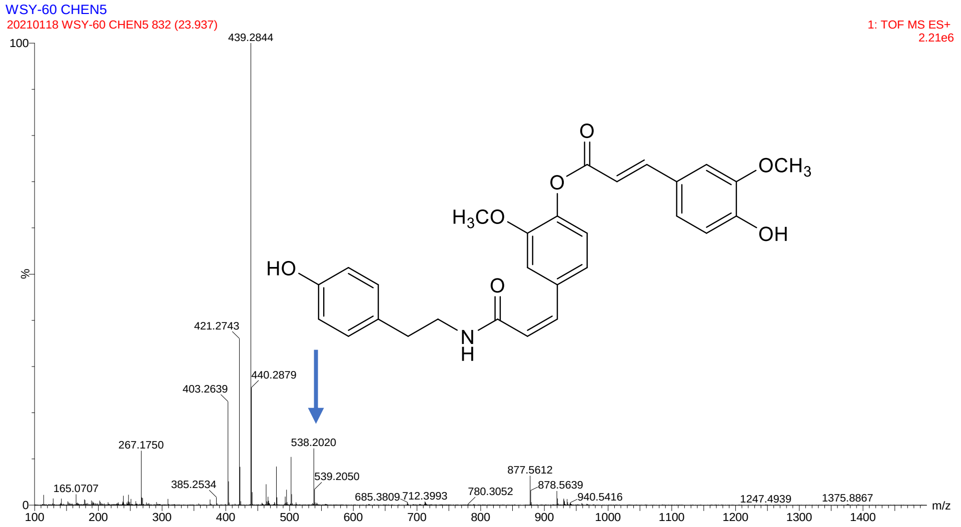

Figure S31. Kruskal-Wallis analysis results for each group

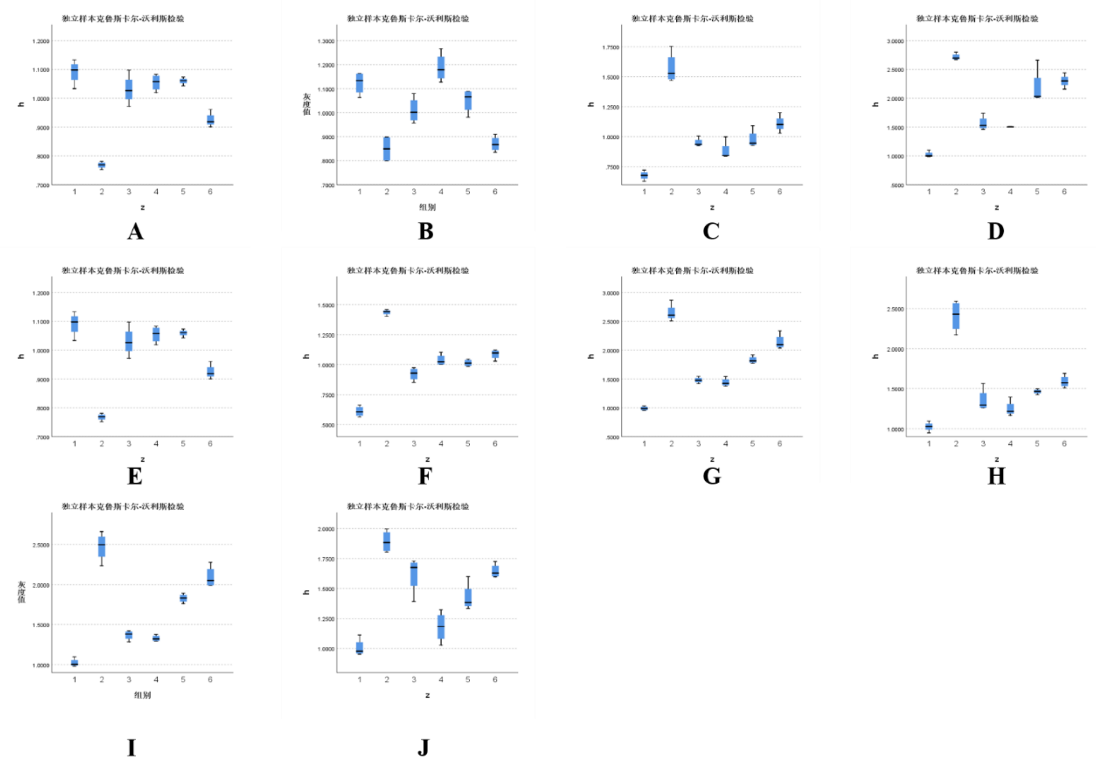

Kruskal-Wallis analysis results for each group of TREM2 (**A**); Kruskal-Wallis analysis results for each group of DAP12 (**B**); Kruskal-Wallis analysis results for each group of iNOS (**C**); Kruskal-Wallis analysis results for each group of COX-2 (**D**); Kruskal-Wallis analysis results for each group of TLR4 (**E**); Kruskal-Wallis analysis results for each group of MyD88 (**F**); Kruskal-Wallis analysis results for each group of NLRP3 (**G**); Kruskal-Wallis analysis results for each group of Caspase-1 (**H**); Kruskal-Wallis analysis results for each group of IBA-1 (**I**); Kruskal-Wallis analysis results for each group of pTau/Tau (**J**).

Table S1. The results for TREM2 of Kruskal-Wallis ANOVA

Group	Median [*P25*, *P75*]	H	*P*
Normal	1.10 [1.04, 1.12]		
Model**	0.76 [0.75, 0.77]		
DPZ##	1.03 [0.98, 1.08]	17.94	0.003
LDS (400 μg/mL) ##	1.06 [1.02, 1.08]		
LDS (200 μg/mL) ##	1.05 [1.04, 1.07]		
LDS (100 μg/mL) ##	0.91 [0.90, 0.95]		

Table S2. The results for Dap12 of Kruskal-Wallis ANOVA

Group	Median [*P25*, *P75*]	H	*P*
Normal	1.13 [1.07, 1.16]		
Model **	0.84 [0.79, 0.89]		
DPZ ##	1.00 [0.96, 1.07]	20.12	0.001
LDS (400 μg/mL) ##	1.18 [1.13, 1.25]		
LDS (200 μg/mL) ##	1.06 [0.99, 1.09]		
LDS (100 μg/mL) ##	0.86 [0.84, 0.90]		

Table S3. The results for iNOS of Kruskal-Wallis ANOVA

Group	Median [*P25*, *P75*]	H	*P*
Normal	0.67 [0.64, 0.71]		
Model **	1.53 [1.47, 1.71]		
DPZ ##	0.94 [0.93, 0.99]	20.40	0.001
LDS (400 μg/mL) ##	0.84 [0.84, 0.96]		
LDS (200 μg/mL) ##	0.94 [0.93, 1.06]		
LDS (100 μg/mL) ##	1.10 [1.05, 1.17]		

Table S4. The results for COX-2 of Kruskal-Wallis ANOVA

Group	Median [*P25*, *P75*]	H	*P*
Normal	1.00 [0.99, 1.08]		
Model **	2.70 [2.67, 2.78]		
DPZ ##	1.53 [1.47, 1.69]	21.32	0.002
LDS (400 μg/mL) ##	1.50 [1.49, 1.51]		
LDS (200 μg/mL) #	2.03 [2.01, 2.51]		
LDS (100 μg/mL)	2.30 [2.19, 2.41]		

Table S5. The results for TLR4 of Kruskal-Wallis ANOVA

Group	Median [*P25*, *P75*]	H	*P*
Normal	1.00 [0.98, 1.01]	21.50	0.007
Model **	1.80 [1.79, 1.91]		
DPZ $^{\#\#}$	1.24 [1.22, 1.90]		
LDS (400 μg/mL) $^{\#\#}$	1.10 [1.01, 1.17]		
LDS (200 μg/mL) $^{\#\#}$	1.24 [1.21, 1.28]		
LDS (100 μg/mL) $^{\#}$	1.64 [1.68, 1.55]		

Table S6. The results for MyD88 of Kruskal-Wallis ANOVA

Group	Median [*P25*, *P75*]	H	*P*
Normal	0.61 [0.57, 0.65]	20.87	0.002
Model *	1.44 [1.41, 1.45]		
DPZ $^{\#}$	0.93 [0.86, 0.97]		
LDS (400 μg/mL) $^{\#\#}$	1.02 [1.00, 1.09]		
LDS (200 μg/mL) $^{\#}$	1.01 [0.98, 1.04]		
LDS (100 μg/mL)	1.10 [1.04, 1.12]		

Table S7. The results for NLRP3 of Kruskal-Wallis ANOVA

Group	Median [*P25*, *P75*]	H	*P*
Normal	0.99 [0.96, 1.03]	21.93	0.001
Model **	2.61 [2.53, 2.80]		
DPZ $^{\#\#}$	1.48 [1.43, 1.53]		
LDS (400 μg/mL) $^{\#\#}$	1.42 [1.38, 1.52]		
LDS (200 μg/mL) $^{\#\#}$	1.81 [1.78, 1.90]		
LDS (100 μg/mL) $^{\#}$	2.10 [1.04, 2.28]		

Table S8. The results for Caspase-1 of Kruskal-Wallis ANOVA

Group	Median [*P25*, *P75*]	H	*P*
Normal	1.03 [0.97, 1.08]	20.83	0.003
Model **	2.43 [2.21, 2.58]		
DPZ $^{\#\#}$	1.29 [1.26, 1.50]		
LDS (400 μg/mL) $^{\#\#}$	1.22 [1.18, 1.35]		
LDS (200 μg/mL) $^{\#\#}$	1.46 [1.43, 1.49]		
LDS (100 μg/mL) $^{\#}$	1.57 [1.52, 1.67]		

Table S9. The results for IBA-1 of Kruskal-Wallis ANOVA

Group	Median [*P25*, *P75*]	H	*P*
Normal	1.00 [0.98, 1.07]		
Model **	2.49 [2.29, 2.63]		
DPZ ##	1.38 [1.30, 1.42]	21.70	0.001
LDS (400 μg/mL) ##	1.32 [1.29, 1.36]		
LDS (200 μg/mL) ##	1.83 [1.76, 1.88]		
LDS (100 μg/mL) #	2.05 [2.00, 2.23]		

Table S10. The results for p-Tau/Tau of Kruskal-Wallis ANOVA

Group	Median [*P25*, *P75*]	H	*P*
Normal	0.98 [0.95, 1.08]		
Model **	1.88 [1.81, 1.98]		
DPZ #	1.46 [1.68, 1.72]	20.96	0.001
LDS (400 μg/mL) ##	1.18 [1.05, 1.30]		
LDS (200 μg/mL) #	1.38 [1.34, 1.55]		
LDS (100 μg/mL) #	1.63 [1.60, 1.71]		

***P < 0.05, **P < 0.01 vs. Control group; #P < 0.05, ##P < 0.01 vs. Model group.**
